# Targeting Astrocyte Signaling Alleviates Cerebrovascular and Synaptic Function Deficits in a Diet-Based Mouse Model of Small Cerebral Vessel Disease

**DOI:** 10.1523/JNEUROSCI.1333-22.2023

**Published:** 2023-03-08

**Authors:** Pradoldej Sompol, Jenna L. Gollihue, Blaine E. Weiss, Ruei-Lung Lin, Sami L. Case, Susan D. Kraner, Erica M. Weekman, John C. Gant, Colin B. Rogers, Dana M. Niedowicz, Tiffany L. Sudduth, David K. Powell, Ai-Ling Lin, Peter T. Nelson, Olivier Thibault, Donna M. Wilcock, Christopher M. Norris

**Affiliations:** ^1^Sanders–Brown Center on Aging; ^2^Departments of Pharmacology and Nutritional Sciences; ^3^Physiology; ^4^Neuroscience; ^5^Pathology, University of Kentucky College of Medicine, Lexington, Kentucky 40536

**Keywords:** Alzheimer's disease, Ca^2+^, neurovascular coupling, reactive astrocytes, synapses, vascular dementia

## Abstract

Despite the indispensable role that astrocytes play in the neurovascular unit, few studies have investigated the functional impact of astrocyte signaling in cognitive decline and dementia related to vascular pathology. Diet-mediated induction of hyperhomocysteinemia (HHcy) recapitulates numerous features of vascular contributions to cognitive impairment and dementia (VCID). Here, we used astrocyte targeting approaches to evaluate astrocyte Ca^2+^ dysregulation and the impact of aberrant astrocyte signaling on cerebrovascular dysfunction and synapse impairment in male and female HHcy diet mice. Two-photon imaging conducted in fully awake mice revealed activity-dependent Ca^2+^ dysregulation in barrel cortex astrocytes under HHcy. Stimulation of contralateral whiskers elicited larger Ca^2+^ transients in individual astrocytes of HHcy diet mice compared with control diet mice. However, evoked Ca^2+^ signaling across astrocyte networks was impaired in HHcy mice. HHcy also was associated with increased activation of the Ca^2+^/calcineurin-dependent transcription factor NFAT4, which has been linked previously to the reactive astrocyte phenotype and synapse dysfunction in amyloid and brain injury models. Targeting the NFAT inhibitor VIVIT to astrocytes, using adeno-associated virus vectors, led to reduced GFAP promoter activity in HHcy diet mice and improved functional hyperemia in arterioles and capillaries. VIVIT expression in astrocytes also preserved CA1 synaptic function and improved spontaneous alternation performance on the Y maze. Together, the results demonstrate that aberrant astrocyte signaling can impair the major functional properties of the neurovascular unit (i.e., cerebral vessel regulation and synaptic regulation) and may therefore represent a promising drug target for treating VCID and possibly Alzheimer's disease and other related dementias.

**SIGNIFICANCE STATEMENT** The impact of reactive astrocytes in Alzheimer's disease and related dementias is poorly understood. Here, we evaluated Ca^2+^ responses and signaling in barrel cortex astrocytes of mice fed with a B-vitamin deficient diet that induces hyperhomocysteinemia (HHcy), cerebral vessel disease, and cognitive decline. Multiphoton imaging in awake mice with HHcy revealed augmented Ca^2+^ responses in individual astrocytes, but impaired signaling across astrocyte networks. Stimulation-evoked arteriole dilation and elevated red blood cell velocity in capillaries were also impaired in cortex of awake HHcy mice. Astrocyte-specific inhibition of the Ca^2+^-dependent transcription factor, NFAT, normalized cerebrovascular function in HHcy mice, improved synaptic properties in brain slices, and stabilized cognition. Results suggest that astrocytes are a mechanism and possible therapeutic target for vascular-related dementia.

## Introduction

As a central component of the neurovascular unit, astrocytes regulate cerebral blood flow, vascular integrity, brain metabolism, and neuronal network activity and plasticity, all things that are compromised in Alzheimer's disease (AD), where signs of astrocyte reactivity are profound and widespread (Price et al., 2021). In postmortem human AD brain specimens and rodent models of AD, reactive astrocytes exhibit signs of Ca^2+^ dysregulation (Kuchibhotla et al., 2009) or hyperactivation of key signaling mediators including the Ca^2+^/calmodulin-dependent protein phosphatase calcineurin (CN) and the CN-dependent transcription factor nuclear factor of activated T cells (NFAT; Norris et al., 2005; Abdul et al., 2009; Lim et al., 2013; Pleiss et al., 2016; Sompol et al., 2017). Drugs that target CN/NFAT activity including commercially available CN inhibitors (e.g., tacrolimus), small chemical NFAT inhibitors (e.g., Q134R), or peptide-based NFAT inhibitors (e.g., VIVIT) improve neuronal viability and/or function in mouse, rat, and canine models of AD-like pathology (Dineley et al., 2007; Yoshiyama et al., 2007; Taglialatela et al., 2009; Rozkalne et al., 2011; Furman et al., 2012; Hudry et al., 2012; Arora et al., 2013; Cavallucci et al., 2013; Sompol et al., 2017; Radhakrishnan et al., 2021; Sompol et al., 2021). Beneficial actions in brain are found even when CN/NFAT inhibition is limited to astrocytes (Furman et al., 2012; Sompol et al., 2017), suggesting that reactive astrocytes play a causative role in pathophysiology and cognitive decline in AD.

In addition to AD, dementia can arise from a variety of other pathologies accompanied by astrocyte reactivity. Vascular contributions to cognitive impairment and dementia (VCID) is the second leading cause of dementia, behind AD, and is the most common comorbidity found with AD and other AD-related dementias (ADRDs). Reactive astrocytes in rodent models of VCID exhibit several phenotypic features in common with astrocytes in AD models, including Ca^2+^ dysregulation (Winship and Murphy, 2008; Ding et al., 2009; Fordsmann et al., 2019). Additionally, reactive astrocytes associated with cerebrovascular pathology in humans can express high levels of hyperactive CN (Pleiss et al., 2016). However, very few studies have investigated the functional impact of reactive astrocyte signaling, including Ca^2+^ dyshomeostasis, on neural function in VCID models.

Here, we characterized the impact of astrocyte signaling in a diet-based model of hyperhomocysteinemia (HHcy), which exhibits several hallmark features of VCID including vascular inflammation, reduced cerebral blood flow, and cognitive impairment (Hofmann et al., 2001; Sudduth et al., 2013, 2017; Braun et al., 2019; Seaks et al., 2022). Astrocyte Ca^2+^ transients and astrocyte network activity was perturbed in fully awake mice treated with HHcy diet. Moreover, selective inhibition of astrocyte NFAT signaling with adeno-associated virus (AAV) vectors expressing the VIVIT peptide ameliorated progressive astrocyte reactivity and improved several indices of neural function in HHcy diet mice including neurovascular coupling, synaptic function and plasticity, and cognition. The results suggest that reactive astrocytes, and aberrant CN/NFAT activity in particular, are a common feature of AD and VCID that could be exploited to treat a broad swath of dementia cases.

## Materials and Methods

### Animals

C57BL/6J mice (7–8 weeks old) both males and females were purchased from The Jackson Laboratory. Mice were housed in standard laboratory cages under 12 h light/dark cycles in a pathogen-free environment in accordance with University of Kentucky guidelines. Mice were placed in a reversed dark/light cycle room 7 d before cranial window surgery and had access to food and water *ad libitum*. All animal procedures were conducted in accordance with the National Institutes of Health *Guide for the Care and Use of Laboratory Animals* and were approved by University of Kentucky Institutional Animal Care and Use Committees.

### AAV vectors

cDNAs for EGFP control and VIVIT-EGFP (to inhibit NFAT signaling) were encoded in AAV2/5 vectors downstream of the human GFAP promoter (Gfa2) as described in our previous work (Furman et al., 2012, 2016; Sompol et al., 2017). AAV-Gfa2 vectors (purchased from the University of Pennsylvania Viral Vector Core) drive transgene expression selectively in astrocytes with no transgene expression observed in nonastrocyte cell types (Furman et al., 2012, 2016). Mice were injected (into barrel cortex or hippocampus, see below) with AAV2/5-Gfa2 vectors at 2 months of age. The four CN-dependent NFAT isoforms exhibit distinct and overlapping functions. VIVIT inhibits multiple NFAT isoforms, including NFAT4 (Aramburu et al., 1999), and can therefore negate compensatory changes in NFAT expression resulting from knockout of any single isoform. For Ca^2+^ imaging studies, mice received barrel cortex injections of AAV5-Gfa104-Lck-GCamp6F vectors to express the genetically encoded Ca^2+^ indicator, GCaMP6F, selectively in astrocytes (viral prep #52924-AAV5, Addgene; gift from Baljit Khakh).

### Diet for inducing HHcy

At ∼1 month after injection of either AAV2/5-Gfa2-EGFP or VIVIT-EGFP vectors, mice were randomly assigned to one of two diet conditions, the HHcy diet or control (CT) diet. The HHcy diet (catalog #TD.130867, Harlan Teklad) is enriched in methionine, but contains low levels of folate, vitamin B6, and vitamin B12. The CT diet (5001 Envigo) was nutritionally matched to the HHcy diet but had normal levels of methionine, folate, B6, and B12. Mice were maintained on the diet for at least 12 weeks (12–15 weeks) and were weighed periodically to ensure that no significant malnourishment was associated with the HHcy diet. Previous work from our labs reported that HHcy diet leads to plasma homocysteine levels in the range of 80–85 µmol/l, which is considered to be representative of moderate HHcy (Sudduth et al., 2013). Additionally, we have shown that adult mice (∼3 months-old) fed with HHcy diet over 12–15 weeks exhibit several phenotypes observed with VCID including vascular inflammation and pathology, reduced cerebral perfusion, astrocyte abnormalities, and cognitive decline (Sudduth et al., 2013, 2017; Braun et al., 2019). Although it would be interesting to investigate the effects of the HHcy diet at advanced ages when dementia is more prominent, we have found that older mice are less tolerable of HHcy diet, resulting in high attrition rates.

### Surgeries for AAV injection and cranial window installation

We followed and modified our surgical techniques according to previous studies (Shih et al., 2012; Goldey et al., 2014; Sompol et al., 2017; Fu et al., 2020). Briefly, mice were placed in an induction box and anesthetized with 2.5% isoflurane (SomnoSuite, Kent Scientific). Mice were then stabilized in a stereotaxic frame and maintained on isoflurane inhalation (1.5–2%, via nose cone) during surgery. The injection site over barrel cortex was marked from the bregma with the coordinates of −1.5 mm AP, ± 3 mm ML. The AAV injection site was used as a center point to create a 3 mm circle for subsequent craniotomy. The injection site and cranial window outline were drilled entirely through the bone with care not to damage the brain surface. AAV vectors (EGFP, VIVIT, or GCaMP6) were loaded (at 10^12^ infectious units per mL) into a Hamilton syringe, which was then mounted to a microinjector (Stoelting). AAV was vertically delivered into the brain (200–300 µm dorsoventral relative to the brain surface) at a rate of 0.2 µl/min (total 2–4 µl). After injection, the needle remained in place for ∼2 min and then was slowly removed from the injection site. The circle bone flap was removed and replaced with a glass cranial window (made from combining 3 and 4 mm glass coverslips with optical glue and UV light curing). The glass window was stabilized, and the glue was applied surrounding the glass window. Once the glue was dry, the head mount (Panda Lab) was attached and glued to the skull and finally secured with dental cement. Analgesic was injected and animals were recovered from anesthesia and returned to home cages.

### Two-photon imaging in awake mice

Mice were anesthetized and placed on the imaging platform of a Scientifica Hyperscope, powered by a fixed (1045 nm) and tunable Insight X3 laser (Spectra-Physics), range from 680 to 1300 nm, and equipped with a 16×, 0.8 NA, 3 mm working distance objective lens (Nikon Instruments) and GaAsP photomultipliers (Hamamatsu Photonics). MATLAB (MathWorks) was used to drive ScanImage acquisition software (Vidrio Technologies). Cranial windows were aligned underneath the objective lens, and mice received retro-orbital injections of fluorescein-dextran or rhodamine-dextran dye (40 kDa, 5% w/v in saline) to visualize blood vessels. Anesthesia was then discontinued, and all subsequent imaging was performed on fully awake mice.

### Two-photon imaging of astrocyte Ca^2+^ transients

Immediately before cranial window installation, some mice received an intracranial injection of AAV5-Gfa104-lck-GCaMP6f and were imaged between 3–4 weeks later. At the beginning of the imaging session, GCaMP6 fluorescence (920 nm excitation wavelength) was monitored in barrel cortex astrocytes at a depth of ∼50 µm. A 10 min baseline was recorded in 180 × 180 µm FOVs to assess spontaneous Ca^2+^ signaling properties. After the baseline, mice received a series of air-puff trials to assess evoked Ca^2+^ activity in astrocytes. Air-puff trains (10 Hz, 1 psi, 10 s) were delivered to the contralateral vibrissae, and Ca^2+^ changes were monitored before, during, and for 30 s after stimulation. A threshold analysis was used to extract Ca^2+^ events from the raw trace of each region of interest (ROI). ROIs are defined from the full frame averages in a time series acquisition using a MATLAB routine composed of a local maximum measurement (16-bit grayscale), a size filter (600–6000 pixels), and sensitivity adjustments. All ROIs were checked for the presence of typical astrocyte morphology properties. The threshold for an event in each ROI was derived from the average of the baseline period before the air puff. Events from each ROI were extracted from full time series data using a peak detection function. After selecting prevalent Ca^2+^ events among ROIs, these time signatures were used to filter time series data of each ROI for moments of Ca^2+^ transience. From these extracted traces, the amplitude, rise time, and decay time of GCAMP6 fluorescence were measured. Each trace was normalized to prepeak baselines (%ΔF/F). In some mice, GCaMP6 fluorescence was also assessed in perivascular ROIs (i.e., end feet) in relation to blood vessel dilations. Blood vessels were visualized using retroorbital injected rhodamine-dextran as described below for neurovascular coupling experiments. For correlogram measures of astrocyte network activity (see Fig. 2*A*,*D*) binarized events were used to calculate a correlation coefficient (CC) for each pair of ROIs per mm^2^. Analyses were limited to coactive ROIs (ROIs that showed simultaneous Ca^2+^ transients) and only to those FOVs with >100 correlated ROIs per mm^2^. A weighted CC for each ROI pair was determined by multiplying the CC by the ratio between the number of detected events per ROI pair and the total number of samples (2 Hz × 60 s = 120 samples per ROI; total of 240 samples for each ROI pair). Only those ROI pairs with a weighted CC value >0.02 were included in statistical analyses. Each dot in the correlogram (see Fig. 2*A*,*D*) represents the position of an ROI in the FOV, where the size of the dot indicates the sum of all weighted CCs with other ROIs. The color of the links (line) between each pair of ROIs represents the CC for that pair (see Fig. 2*D*, color map on far right; note the cutoff range is 0.4–1). The average number of coactive connections per mm^2^ in each FOV and the distance between coactive ROIs (average distance in micrometers between each pair along the *X*, *Y* plane) were compared across CT and HHcy diet conditions.

### NFAT4 expression in astrocytes

Forty-micrometer free-floating sections were immunostained for NFAT4 and GFAP, as described (Sompol et al., 2021). Sections were washed in 1× PBS plus 0.1% Triton X-100 for 3× 10 min. Peroxidase background was blocked using 3% peroxide in methanol for 30 min at room temperature. Nuclei were stained using 1:500 DAPI (catalog #62248, Thermo Fisher Scientific) for 10 min incubation. Sections were again washed, then blocked for 30 min at room temperature to remove background (0.30 × g BSA/final ml in 1× PBS/0.1% Triton X-100). Primary antibodies were applied overnight at 4°C (1:75 NFAT4; catalog #SC-8405 Santa Cruz Biotechnology; 1:200 GFAP; catalog #12389S, Cell Signaling Technology), diluted to proper concentration using blocking buffer. The next day, sections were washed, and the following secondary antibodies were applied: anti-rabbit 594 (1:200; catalog #A11072, Invitrogen) and goat anti-mouse HRP (TSA 488, catalog #B40912, Invitrogen) overnight at room temperature on a rotator. The sections were again washed and incubated in the Tyramide superboost system according to manufacturer protocol (TSA 488, catalog #B40922, Invitrogen) for 10 min at room temperature. The sections were washed one last time before mounting on slides and coverslipped using ProLong Diamond Antifade Mountant (Invitrogen). For representative images demonstrating 3D colocalization of NFAT4, DAPI, and GFAP, z-stack images were first captured on a Nikon confocal Tie2 microscope using NIS-Elements software (Nikon) at 60× magnification. The resulting z-stack image was then uploaded into Imaris (Oxford Instruments). Layers were created for each individual channel to mask the positive signal of each fluorophore and applied to each level of the z-stack image. The resulting 3-D image was then rotated to show the internal colocalization of NFAT4 and DAPI.

### NFAT4 DNA binding activity

Electrophoretic mobility shift assays (EMSAs) were used to quantify NFAT4-DNA binding activity. NFAT nucleotide binding probes, antibodies, and all procedures were nearly identical to those described in our previous work (Furman et al., 2016). Single brain hemispheres from mice treated with CT or HHcy diet were flash frozen and stored at −80°C until use. Whole-cell extracts from frozen tissue were prepared using a kit from Active Motif following the instructions of the manufacturer. Protein concentrations were determined using a Bradford assay. Fluorescent NFAT binding probes (10 femtomoles) containing the nucleotide sequence TGGAAAAT (from the IL-4 NFAT binding site) were used as DNA probes in a reaction buffer containing 10 mm Tris, pH 7.5, 50 mm NaCl, 1 mm EDTA, 1 mm DTT, 0.05% NP40, 5% glycerol, 0.5 µg poly[d(IC), 5 µg BSA, and 10% 10× orange loading buffer (LI-COR Biosciences), 1% protease inhibitor cocktail III (EMD Millipore), and 1% phosphatase inhibitor cocktail II (EMD Millipore). For quantitative comparisons, extracts from four control and four HHCy samples were analyzed on the same gel, and a sample of these is shown (see Fig. 3*C*), and the quantitative comparison of all samples is shown (see Fig. 3*D*). An example of an EMSA with competition assays, either the wild-type sequence or a mutant competitor containing an altered NFAT binding site (TGGAAAA→CTTTAAA) and supershift/block shifts induced with NFAT1-4 antibodies is shown in Extended Data Figure 3-1. Primary antibodies (used individually) were the following: NFAT1 (catalog #ab2272, Abcam), NFAT2 (catalog #sc-13033x, Santa Cruz Biotechnology), NFAT3 (catalog #sc-13036x, Santa Cruz Biotechnology), and NFAT4 (catalog #sc-8405x, Santa Cruz Biotechnology). Reactions and subsequent electrophoresis were conducted as described previously (Furman et al., 2016). Gel imaging was performed on an Odyssey scanner (LI-COR) and analyzed with Image Studio 2.1 software (LI-COR).

### CN protein levels

CN levels were assessed in nuclear and cytosolic fractions as described in our earlier work (Mohmmad Abdul et al., 2011; Sompol et al., 2017). Half brains from CT and HHcy diet mice were harvested and stored at −80°C as described above. Cytosolic and nuclear homogenate samples were resolved via SDS PAGE on 4–20% Criterion gradient gels (Bio-Rad) and then transferred to Immobilon-FL PVDF membranes (Millipore Sigma). Membranes were probed for calcineurin anti-CN-Aα (catalog #07-1492, EMD Millipore), which tags the N-terminal region of CN and identifies both full-length phosphatase and C-terminal proteolized phosphatase fragments (Mohmmad Abdul et al., 2011). GAPDH (catalog #ab9484, Abcam) and H3 Histone (catalog #H0164, Sigma-Aldrich) were used to mark cystolic versus nuclear fractions, and βActin (catalog #3700S, Cell Signaling Technology) was used to normalize CN signal as an internal control. Membranes were then imaged on an Odyessey Scanner (LI-COR) and the quantification conducted using Image Studio 2.1 software (LI-COR).

### Two-photon imaging of EGFP volume in astrocytes

Mice received injections of AAV-Gfa2-EGFP and AAV-Gfa2-VIVIT-EGFP vectors into barrel cortex followed by installation of a glass cranial window and head holder. At ∼1 month after injection, mice were anesthetized and two-photon microscopy was used to image barrel cortex astrocytes (at a depth up to 50–100 µm) before the initiation of HHcy diet. After the prediet imaging session, mice were returned to their home cages and were started on HHcy diet or were continued on CT diet. In a pilot study, EGFP levels were investigated at 1 month postdiet induction, but no significant diet/treatment effects were observed (data not shown). All subsequent measures were therefore performed at 3 months postdiet induction, a time point associated with maximal changes in other HHcy-sensitive phenotypes including astrocyte end foot disruption (Sudduth et al., 2017), reduced cerebral blood flow (Braun et al., 2019), and cognitive impairment (Sudduth et al., 2013). Astrocyte-expressed EGFP images were acquired with a 16× objective in a 4× digital zoom with Z-series (1 µm steps). Three-dimension image rendering and analyses were performed via NIS-Elements software. EGFP volume from the entire FOV (µm^3^) in barrel cortex was calculated per mouse and compared between prediet and 3-month postdiet conditions.

### Collagen IV labeling and microvessel size histograms

For microvessel immunohistochemical labeling shown in Extended Data Figure 4-1, free-floating coronal sections (40 µm thickness) were stained for blood vessels (collagen IV). Peroxidase background was blocked with a 30 min incubation in room temperature 3% peroxide in methanol. The sections were washed for 5 min in water, then 2 × 5 min in 1× TBS plus 0.1% Triton X-100. Antigen retrieval was performed in citrate buffer pH 9 (catalog #K8000, Dako) at 80°C for 10 min. Sections were washed in TBS/Triton X, then background was blocked by incubating in blocking buffer (TBS/Triton X plus 5% goat serum plus 0.03 × g BSA/final ml) for 1 h at room temperature. Sections were then incubated in primary antibody overnight at 4°C (1:1000; Collagen IV, catalog #ab236640, Abcam) diluted in blocking buffer. The sections were washed in TBS/Triton X, then incubated in secondary antibody for 1 h at room temperature (goat anti-rabbit, catalog #BA-1000, Vector). The sections were washed, then incubated in ABC solution (catalog #PK-6100, Vector) for 1 h, followed by another wash. The sections were then stained using DAB (catalog #SK-4100, Vector), quenched in water, then washed. Finally, the sections were mounted onto slides and dried overnight. The slides were then sequentially dehydrated in ethanol and finally in SafeClear (catalog #23-314629, Fisher Scientific) before coverslipping using DPX mounting medium (catalog #100503-834, VWR).

For vessel size distribution analysis, slides were imaged at 20× using Nikon BioPipeline imaging system. ROIs were drawn around the hippocampus (Nikon NIS-Elements software), and the width of each vessel within the ROI was recorded. The vessels were then binned according to width (0.5 µm intervals) to find the frequency of each width within equal-sized ROIs. Histograms were created using GraphPad software, and Kolmogorov–Smirnov tests were used to determine statistically significant differences between groups.

### Two-photon imaging of cerebral vessel leakiness

Mice received AAV injections (AAV-Gfa2-EGFP and AAV-Gfa2-VIVIT) into barrel cortex and were fitted with glass cranial windows as described above. At 3 months postdiet induction, mice were anesthetized using isoflurane, placed on a heating pad, and affixed to the imaging stage using their head mount. Mice were maintained under isoflurane for the duration of the imaging session. Once placed on the stage, mice were given an intravenous injection of 50 µl of 40 kDa rhodamine-dextran diluted in saline. Using an 850 nm wavelength, both astrocytes (EGFP) and vessels (rhodamine) were imaged, and a 300 µm z-stack (1 µm steps) was taken at 0 min (directly after injection of rhodamine-dextran) and then 15, 30, 45, and 60 min after injection. Three-dimensional image rendering and analyses were performed in NIS-Elements software. Rhodamine and EGFP mean intensity were measured in the entire FOV, and a rhodamine/EGFP ratio was calculated at each of the 15 min time points to account for the decrease in signal over time. Then a ratio to the 0 min time point was generated at each time point (15, 30, 45, and 60 min) for each animal.

### Two-photon imaging of arterioles and capillaries

In mice injected with AAV-Gfa2-EGFP or AAV-Gfa2-VIVIT, we identified penetrating arterioles (with rhodamine-dextran) in barrel cortex located among EGFP-expressing astrocytes (850 nm excitation wavelength). Air-puff stimulation of contralateral whiskers was conducted as described above for Ca^2+^ imaging studies (at least three trials per mouse). Images were acquired at two frames per second. Air-puff-dependent changes in arteriole lumen volume were calculated off-line using ImageJ and custom-developed MATLAB software that tracked ROI segments from active arterioles and their change in radius over time. The minimum volume of the arteriole segment was divided against the maximal volume measurement during the recording (before, during, and for 30 s after whisker stimulation). To control against potential artificial drops in volume, because of motion artifact or otherwise, the minimum volume of the arteriole was determined by averaging the lower fifth percentile of the volume measurements. First-order capillaries located ∼50 µm from targeted penetrating arterioles (Rungta et al., 2018) were investigated in separate air-puff trials (interleaved with arteriole trials). Capillaries were scanned along one dimension, bidirectionally with high-speed galvanic motors (10 µm, ∼2000 Hz) to detect red blood cell (RBC) motion. RBC velocity was then calculated using custom MATLAB software that used the radon transform method as described (Drew et al., 2010). Data were binned into 50 ms segments before analysis and was postprocessed by a moving average filter through a window of 750 ms. The maximum change from baseline and the time to maximum velocity were calculated.

### Cerebral blood flow measurement

We measured cerebral blood flow (CBF) using MRI-based arterial spin labeling (ASL) techniques as previously described (Lin et al., 2020). Briefly, MRI experiments were performed on a 7T MRI scanner (Clinscan, Bruker BioSpin) at the Magnetic Resonance Imaging and Spectroscopy Center of the University of Kentucky. Mice were anesthetized with 4.0% isoflurane for induction and then maintained in a 1.2% isoflurane and air mixture using a nose cone. Heart rate (90–110 bpm), respiration rate (50–80 breaths/min), and rectal temperature (37 ± 1°C) were continuously monitored and maintained. A water bath with circulating water at 45–50°C was used to maintain the body temperature. A whole-body volume coil was used for transmission, and a mouse brain surface coil was placed on the top of the head for receiving. We measured CBF using MRI-based pseudo-continuous ASL (pCASL) techniques. Paired control and label images were acquired in an interleaved fashion with a train of Hanning window-shaped radio frequency pulses of duration/spacing = 200/200 µs, flip angle = 25° and slice-selective gradient = 9 mT/m, and a labeling duration = 2100 ms. The images were acquired by 2D multislice spin-echo echoplanar imaging with FOV = 18 × 18 mm2, matrix = 128 × 128, slice thickness = 1 mm, 10 slices, TR = 4000 ms, TE = 35 ms, and 120 repetitions. pCASL images were analyzed with in-house written codes in MATLAB (MathWorks) to obtain quantitative CBF (with units of ml/g per min). Brain structural T2-weighted images were acquired with FOV =18 × 18 mm2, matrix = 256 × 256, slice thickness = 1 mm, 10 slices, repetition time (TR) = 1500 ms, and echo time (TE) = 35 ms. The CBF images were then superimposed to the corresponding structural images using Multi-Image Analysis GUI (Mango) software (http://rii.uthscsa.edu/mango/). Regional analysis was performed to obtain quantitative CBF values from bilateral hippocampus using Mango software.

### Brain slice electrophysiology

Each mouse received an injection of AAV2/5-Gfa2-EGFP control vector into one hippocampus and AAV2/5-Gfa2-VIVIT-EGFP into the hippocampus of the other hemisphere. Thus, the EGFP-expressing hemisphere served as an internal control for VIVIT effects in the other hemisphere. AAV2/5-Gfa2 vectors were injected into 18 mice (4 µl per hemisphere at a rate of 0.2 µl/min), and mice were assigned to CT diet (*n* = 9) or HHcy diet (*n* = 9) conditions as described above. All electrophysiology protocols including slicing conditions, preincubation protocols, recording solutions, and temperatures were identical to our previous work (Mathis et al., 2011). Briefly, brain slices were transferred to an RC-22 chamber (Warner Instruments) on the stage of a Nikon Eclipse E600 microscope and perfused continuously with oxygenated artificial CSF (ACSF; ∼32°C). A bipolar platinum iridium wire was placed in stratum radiatum near the CA1 border to activate CA3 Schaffer collaterals, and a glass micropipette containing a silver chloride wire and filled with ACSF was placed in CA1 stratum radiatum to record presynaptic fiber volleys (FVs) and EPSPs. Synaptic strength curves were generated by taking the ratio of the EPSP slope (mV/ms) to the FV amplitude at each stimulus intensity level (12 levels total). Stimulus intensity, controlled by a World Precision Instruments constant current stimulus isolator was then adjusted to yield an ∼1 mV field potential. A 20 min baseline of EPSPs (collected at 0.033 Hz) was recorded before the delivery of two trains of 100 Hz stimulation (1 s per train, with an intertrain interval of 10 s) to induce long-term potentiation (LTP), followed by a second (post-LTP) baseline period (also collected at 0.033 Hz). Stimulus timing and data acquisition were controlled by a MultiClamp 700B amplifier, Digidata 1330, and pClamp 9 software (Molecular Devices). FV and synaptic strength curves were fit with a three-parameter sigmoidal equation using GraphPad Prism (version 7) software. Curve parameters and other basal field potential properties were extracted and analyzed as described in our earlier work (Norris and Scheff, 2009; Pleiss et al., 2016; Sompol et al., 2021). Parameters included maximal FV amplitude, half-maximal FV, curve slope, maximal EPSP slope, maximal EPSP/FV ratio, and EPSP at population spike threshold (i.e., pop spike threshold). LTP levels were normalized to the pre-100 Hz baseline and averaged across the last 10 min of the post-100 Hz baseline. Synaptic parameters were averaged across slices within each hemisphere and compared across control and VIVIT-treated hemispheres within each mouse.

### Behavior testing

Mice received bilateral hippocampal injections of either AAV2/5-Gfa2-EGFP or AAV2/5-Gfa2-VIVIT-EGFP and were maintained on CT or HHcy diet for 12 weeks. For open field testing, mice were placed in a square Plexiglas box and allowed to move freely for 15 min (Sukoff Rizzo et al., 2018). Animals were monitored by an overhead camera to assess measures of general activity, gross locomotor activity, and exploration habits. For Y maze testing, methods were highly similar to our previous work (Sompol et al., 2021). Each mouse was placed in the center of the maze and allowed to explore uninterrupted for 8 min. An arm entry was counted when all four limbs were within the arm. The number of arm entries and the number of triads were recorded to calculate the percentage of alternations [% Alternation = (Number of Alternations)/(Total number of arm entries − 2) × 100]. Neither AAV nor diet treatment affected the number of total arm entries, and there was no correlation between the number of alternations and the number of arm entries within or across groups.

### Sex as a biologically relevant variable

No sex differences were observed for any of the outcome measures reported here. Males and females were therefore combined within each group for all statistical analyses.

### Statistical analyses

As outlined below in Results, diet and AAV treatment effects on biobehavioral markers were determined using a variety of parametric and nonparametric tests, including *t* tests, ANOVA, and repeated-measures ANOVA (rmANOVA). For two-way ANOVAs, significant AAV by diet interactions were followed by evaluating simple main effects using Sidak's multiple-comparisons test. Simple main effects were examined following rmANOVAs using paired *t* tests. CBF levels were compared between EGFP- and VIVIT-treated HHcy diet mice using a directional *t* test. All statistical comparisons were made with GraphPad Prism version 7 software (RRID:SCR_002798) or StatView version 5 software. Statistical significance for all comparisons was set at *p* ≤ 0.05.

## Results

### HHcy is associated with dysregulation of astrocyte Ca^2+^ signaling

**Figure 1.**
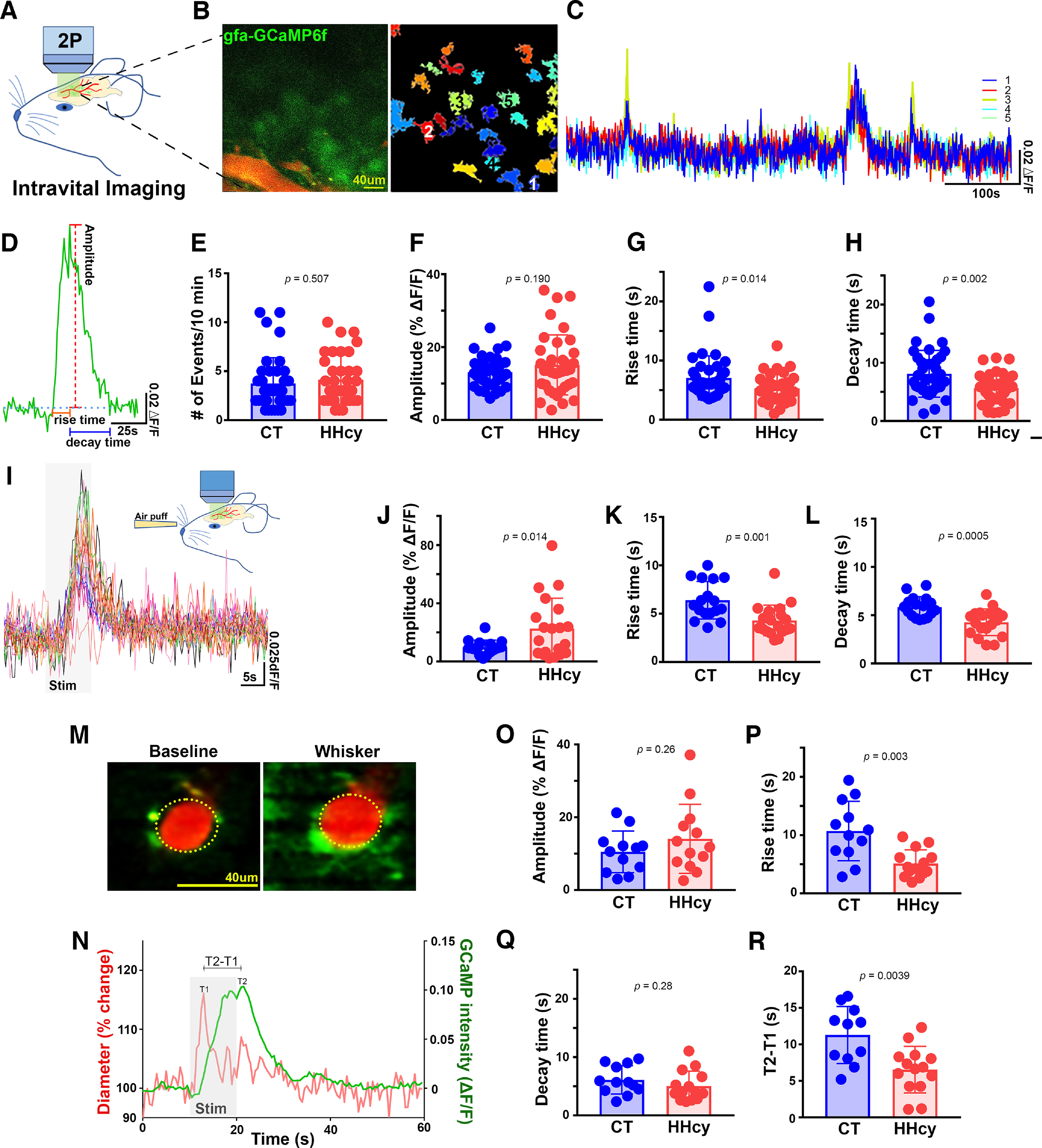
Effects of HHcy diet on spontaneous and evoked Ca^2+^ transients in barrel cortex astrocytes. ***A***, Two-photon imaging was performed through a glass cranial window over barrel cortex in fully awake mice treated for 12 weeks with CT (*n* = 4) or HHcy diet (*n* = 4). ***B***, With the use of AAV-Gfa104-lckGCaMP6f vectors, raw Ca^2+^ fluctuations (changes in green fluorescence) were assessed in astrocytes near rhodamine-labeled cerebrovessels (left) and extracted as pseudo-colored ROIs via software-based detection (right). Scale bar, 40 µm. ***C***, representative spontaneous Ca^2+^ transients (ΔF/*F*) recorded in multiple ROIs in the representative FOV shown in ***B***. ***D***, Representative trace illustrating spontaneous Ca^2+^ transient amplitude and rise/decay time measures. ***E–H***, Effects of HHcy on spontaneous Ca^2+^ transient parameters (averaged across ROI in each FOV) including transient frequency (***E***), transient amplitude (***F***), rise time (***G***), and decay time (***H***); *n* = 39 FOVs in CT diet mice; *n* = 37 FOVs in HHcy mice. ***I***, representative Ca^2+^ transients evoked in multiple ROIs of the barrel cortex by air-puff stimulation of the contralateral vibrissae (10 Hz for 10 s). ***J–L***, Effects of diet on amplitude, rise, and decay parameters of evoked Ca^2+^ transients (averaged across ROIs in each FOV). HHcy was associated with greater transient amplitudes (***J***) with faster rise (***K***) and decay (***L***) kinetics; *n* = 18 FOVs in CT diet mice; *n* = 20 FOVs in HHcy mice. ***M***, Before (left) and after (right) images of GCaMP fluorescence (green) in an astrocyte end foot immediately adjacent to a dilating arteriole (red). Scale bar, 40 µm. ***N***, Real-time vasodilatory response and adjacent end foot Ca^2+^ transient. Note that the end foot Ca^2+^ transient begins and peaks after the onset and peak of the dilatory response in the adjacent vessel. ***O–Q***, Effects of diet on amplitude (***O***), rise (***P***), and decay (***Q***) parameters of evoked Ca^2+^ transients in astrocyte end feet. Transients exhibited a faster rise time in HHcy diet mice (***P***). ***R***, The time interval between the peak vessel dilatory response (T1) and the end foot Ca^2+^ transient peak (T2; ***N***). End foot Ca^2+^ responses occurred more rapidly in response to nearby vasodilation; *n* = 12 FOVs in CT diet mice; *n* = 14 FOVs in HHcy mice. Data points represent averaged values per FOV. Statistical comparisons made with unpaired *t* tests.

Dysregulation of astrocytic Ca^2+^ signaling has been reported in mouse models of amyloid pathology and acute brain injury (Kuchibhotla et al., 2009; Delekate et al., 2014). To determine whether HHcy, a well known risk factor for VCID, AD, and other ADRDs (Madigan et al., 2016; Price et al., 2018), is similarly associated with astrocyte Ca^2+^ changes, we used two-photon microscopy to evaluate Ca^2+^ transient properties in individual barrel cortex astrocytes and astrocyte networks of fully awake mice after 3 months of treatment with CT diet or HHcy diet (Fig. 1*A*,*B*). Representative images of raw GCaMP6 fluorescence and pseudo-colored ROIs are shown in Figure 1*B*. Each ROI corresponds roughly to a single astrocyte (see above, Materials and Methods). At the beginning of the session, mice were imaged for 10 min to assess basal GCaMP activity (Fig. 1*C*). Traces were extracted for measures of mean transient amplitude (ΔF/F) and transient rise and decay times (Fig. 1*D*). The number of transients was also measured for each ROI and averaged across the FOV. Overall, 76 FOVs were analyzed from four CT diet and four HHcy diet mice and are shown in Figure 1, *E–H*. For statistical comparisons, *n* = the number of FOVs per group. No differences in spontaneous Ca^2+^ transient frequency were observed across diet groups (Fig. 1*E*). However, spontaneous transients in individual ROIs tended to be greater in amplitude (Fig. 1*F*; *p* = 0.19) and exhibited significantly faster rise (Fig. 1*G*; *p* = 0.014) and decay (Fig. 1*H*; *p* = 0.002) kinetics in HHcy diet mice.

To determine whether evoked Ca^2+^ activity was altered in barrel cortex astrocytes as a function of diet treatment, mice received air-puff stimulation (1 psi, 10 Hz for 10 s) to the contralateral vibrissae. Representative thresholded Ca^2+^ activity for selected ROIs in a CT diet mouse before, during, and after air-puff stimulation is shown in Figure 1*I*. Transient amplitude, rise, and decay times were calculated during air-puff stimulation (as described above) from a total of 38 ROIs from the same eight mice described above. The results revealed significantly greater evoked Ca^2+^ transient amplitudes (Fig. 1*J*) in the HHcy group versus the CT diet mice (*p* = 0.01). Astrocyte Ca^2+^ transients in HHcy mice also exhibited significantly shorter rise (Fig. 1*K*; *p* = 0.001) and decay times (Fig. 1*L*; *p* = 0.0005). We also looked at Ca^2+^ transient parameters in astrocyte end feet surrounding cerebrovessels (Fig. 1*M*,*N*) visualized with rhodamine-dextran (26 total ROIs from eight mice). End feet Ca^2+^ transients were marginally (but, not significantly) greater in amplitude in HHcy diet mice (*p* = 0.26) and, again, were characterized by significantly faster (*p* = 0.003) rise times and marginally faster (*p* = 0.26) decay times (Fig. 1*P*,*Q*). Similar to previous work that investigated astrocyte Ca^2+^ transients in awake mice (Tran et al., 2018), we found that astrocyte end feet Ca^2+^ elevations occurred after vasodilation of adjacent blood vessels regardless of diet treatment (Fig. 1*N*). Moreover, the time interval between maximal vessel dilation and the adjacent end foot Ca^2+^ transient peak was significantly shorter (*p* = 0.004) in HHcy diet mice (Fig. 1*R*).

**Figure 2.**
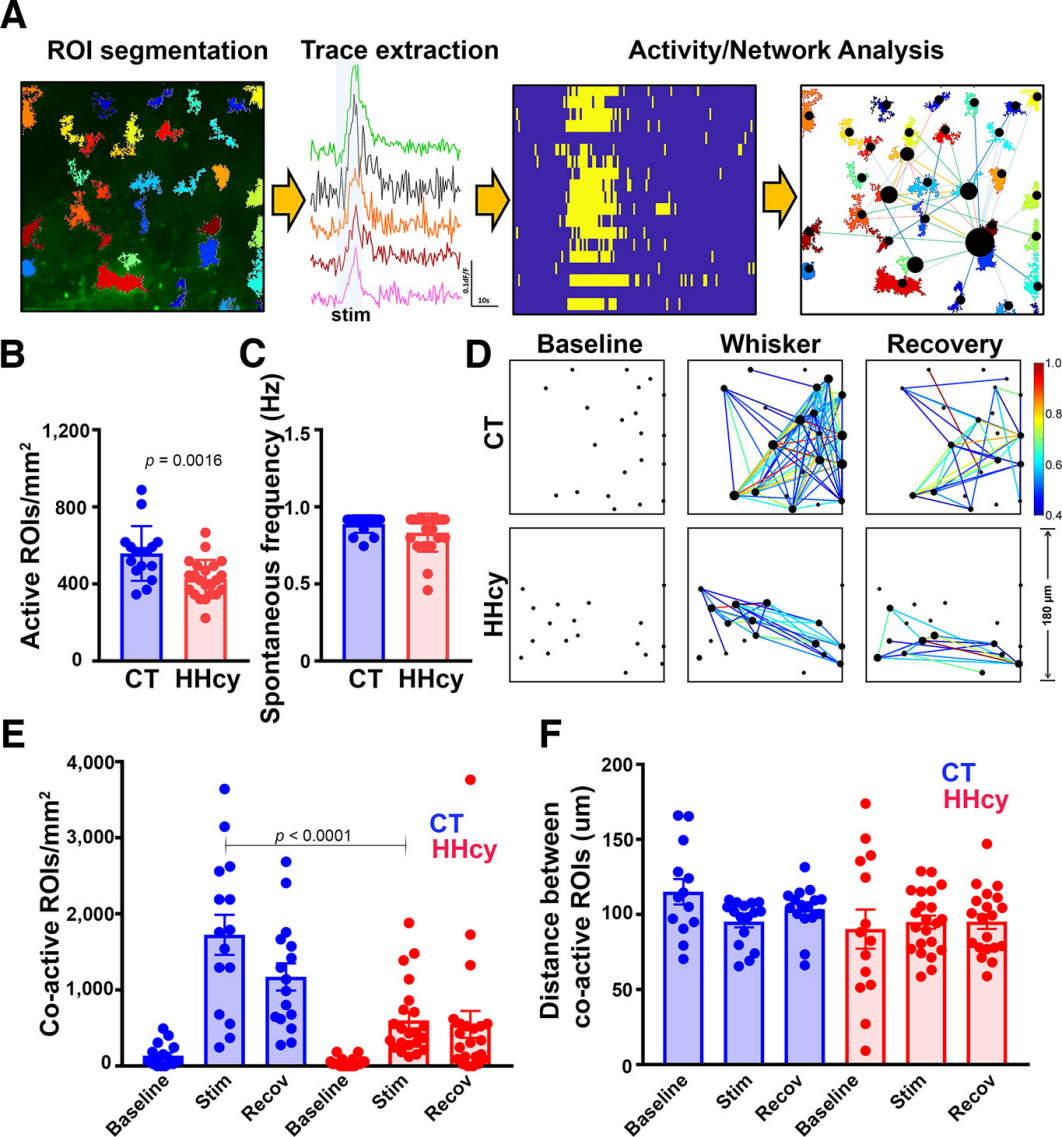
Signal processing and effects of HHcy diet on Ca^2+^ signaling in astrocyte networks. ***A***, ROIs and FOVs from Figure 1 were handled using a customized MATLAB pipeline including the management of ROI segmentation, trace extraction, and activity analysis. ***B***, The number of active ROIs/mm^2^ was significantly reduced in HHcy diet mice, suggesting a reduction in the number of actively signaling astrocytes. ***C***, Average oscillation frequency per ROI (during air-puff stimulation) was not affected by diet treatment. Differences in ***B*** and ***C*** determined with unpaired *t* tests. ***D***, Correlograms from representative FOVs in a CT and a HHcy diet mouse showing coactive astrocyte connections before (baseline), during (whisker), and after (recovery) air-puff stimulation. Each dot represents an ROI/astrocyte in the FOV. The size of each dot represents the sum of weighted correlation coefficients (i.e., correlated activity) with all other astrocytes. The color of the line between astrocytes indicates the unweighted activity correlation. ***E***, Number of coactive ROIs/mm^2^ in mice treated with CT or HHcy diet. The correlated activity in the astrocyte network is low during the baseline but increases significantly during whisker stimulation. Relative to CT diet mice, mice treated with HHcy diet exhibit a smaller increase in the number of coactive ROIs during stimulation. ***F***, The average distance, or length, between coactive ROI pairs during whisker stimulation trials was not affected by diet treatment. Data points in ***B***, ***C***, ***E***, ***F*** represent averaged values per FOV (i.e., *n* = FOVs, 16–18 in CT diet mice, *n* = 18–20 in HHcy mice). Significance determined in ***E*** with Sidak's multiple-comparisons test.

In addition to assessing Ca^2+^ transient properties in individual ROIs, we used a customized MATLAB pipeline including ROI segmentation, trace extraction, and activity analysis (Fig. 2*A*) to quantify the correlated activity in the astrocyte network. The number of active ROIs/FOVs during air-puff stimulation was significantly reduced for the HHcy diet group (*p* = 0.0016; Fig. 2*B*), but the average oscillation frequency per ROI was not sensitive to diet (Fig. 2*C*). Correlated activity among ROI pairs before (baseline), during (stimulation), and after (recovery) air-puff delivery was then calculated for CT and HHcy diet mice. Correlograms from representative FOVs from a CT and HHcy diet mouse are shown in Figure 2*D*, where the size of each dot represents the sum of weighted correlation coefficients with all other ROIs/astrocytes in the FOV. The color of the line between astrocytes indicates the unweighted activity correlation (Fig. 2*D*, color map, far right). A two-way repeated-measures ANOVA was used to assess the effects of diet and stimulation on the number of coactive ROI pairs per mm^2^ (Fig. 2*E*) and the distance between correlated ROI pairs (i.e., CC > 0.4; Fig. 2*F*). The number of coactive ROIs was very low in both diet conditions during the baseline before showing a significant increase during stimulation and recovery (main stimulation effect, *F*_(2,36)_ = 3 0.55, *p* < 0.0001). However, the stimulus-associated increase in coactive ROIs was much greater in the CT diet group versus HHcy diet mice (main diet effect, *F*_(1,37)_ = 17.77, *p* = 0.002). The HHcy group also exhibited shorter distances between correlated ROIs across the entire air-puff trial (main diet effect, *F*_(1,37)_ = 4.43, *p* = 0.042), but path length was not significantly affected by stimulation in either diet treatment group. Together, the results demonstrate complex effects of HHcy on Ca^2+^ signaling properties in astrocytes; individual astrocytes/ROIs in HHcy mice exhibited greater and faster air-puff-associated Ca^2+^ transients compared with CT diet mice (Fig. 1*J*), but correlated activity within the astrocyte network (across multiple ROIs/FOVs) was significantly impaired (Fig. 2*E*) as a result of HHcy treatment.

### HHcy is associated with elevated astrocytic NFAT activation and astrocyte reactivity

**Figure 3.**
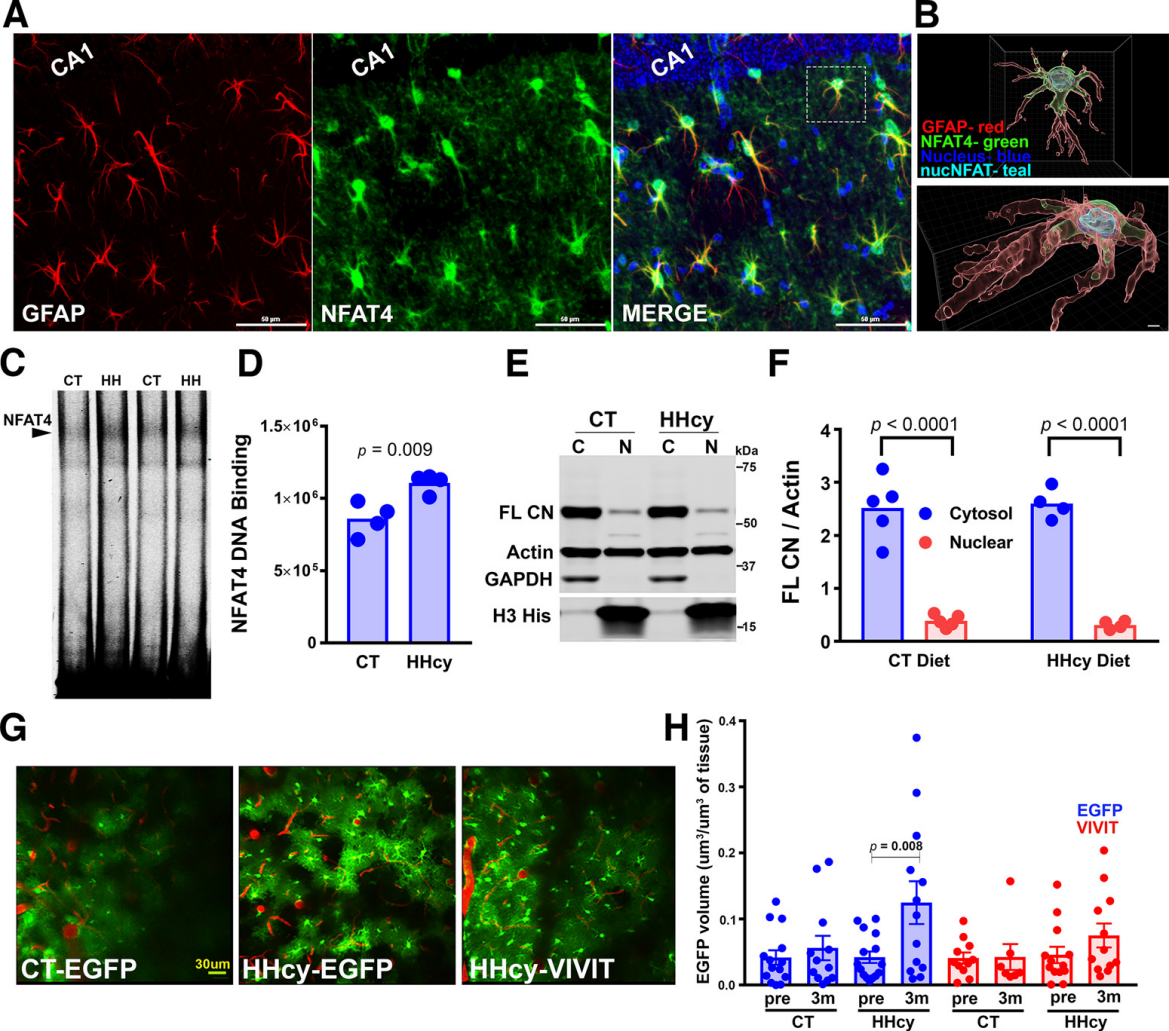
Effects of HHcy diet on CN/NFAT4 properties. ***A***, Confocal micrographs of the hippocampal CA1 region showing strong colocalization of NFAT4 (green) with GFAP-positive (red) astrocytes. Right, Note in the merged image that NFAT4 colocalizes to the nucleus of many GFAP-positive astrocytes, indicating increased NFAT4 activation. Scale, 50 µm. ***B***, Strong nuclear localization of NFAT4 is highlighted in an astrocyte magnified and 3D rendered from the merged image in ***A*** (right, hatched box). Bottom, The same astrocyte at higher magnification. Calibration, 2 µm) and rotated to emphasize the nuclear colocalization of NFAT4 (in teal). ***C***, Representative EMSA from brain tissue harvested from CT diet and HHcy diet mice illustrate NFAT/DNA binding activity. The arrowhead points to bands that are sensitive (i.e., exhibit a block shift) to the addition of a mononclonal NFAT4 antibody (Extended Data Fig. 3-1). ***D***, NFAT4/DNA binding activity was significantly increased in HHcy diet mice. ***E***, Representative WB for the CN Aα subunit in cytosolic and nuclear fractions harvested from brains of CT diet and HHcy diet mice. Note that using an N-terminal antibody, we observed a single band ∼60 kDa in both diet groups, which represents the FL form of CN A. No signs of proteolysis (i.e., bands in the 45–57 kDa range) related to constitutively high CN activity were observed. ***F***, In both diet groups, CN levels were highest in cytosolic fractions, and the nuclear/cytosolic ratio was not affected by diet; *n* = 4–5 mice/group in ***D*** and ***F***. Significance determined with unpaired *t* tests. ***G***, Representative two-photon images showing Gfa2-dependent EGFP expression in barrel cortex astrocytes of individual mice injected with AAV-Gfa2-EGFP or AAV-Gfa2-VIVIT-EGFP vectors. Extended Data Figure 3-2 shows that EGFP volume increases progressively with time on HHcy diet. Images in ***G*** were taken at 3 months postdiet. Scale bar, 30 µm. ***H***, EGFP volume in AAV-Gfa2-EGFP or AAV-Gfa2-VIVIT-EGFP-treated mice at the prediet time point (pre) and after 3 months (m) of CT or HHcy diet (3 months). GFAP promoter-driven EGFP expression in HHcy mice is significantly increased over prediet baseline levels. No significant changes in EGFP levels were observed across time points in CT diet mice or in HHcy mice treated with VIVIT. In ***D***, ***F***, ***H*** each individual data point represents an individual mouse (*n* = 12–13 mice/group). Significance determined with rmANOVA followed by paired *t* tests (pre vs 3m), within diet group.

We next investigated whether altered astrocyte Ca^2+^ signaling is associated with changes in Ca^2+^ signaling pathways related to astrocyte reactivity. The CN/NFAT pathway is highly sensitive to fluctuating intracellular Ca^2+^ levels and shows signs of dysregulation/hyperactivation in multiple neural cell types in the context of neuropathology and cognitive decline (Reese and Taglialatela, 2011; Sompol and Norris, 2018). In astrocytes, changes in CN expression and/or CN-dependent signaling (i.e., NFAT activation) have been linked to reactive astrocyte phenotypes including cellular hypertrophy (Perez-Nievas and Serrano-Pozo, 2018; Sompol and Norris, 2018). Of the four CN-dependent NFAT transcription factors, the NFAT4 isoform shows much greater expression in astrocytes, (relative to neurons and microglia), particularly reactive astrocytes (Serrano-Pérez et al., 2011; Furman et al., 2016; Sompol et al., 2017). Similar to previous work, confocal micrographs (Fig. 3*A*) taken from HHcy mouse brain sections labeled with antibodies to GFAP (red) and NFAT4 (green) confirmed highly selective NFAT4 labeling in astrocytes. Many of the astrocytes in HHcy diet mice exhibited extensive localization of NFAT4 to DAPI-labeled nuclei (Fig. 3*A*,*B*, merged image in *A*), consistent with NFAT4 activation. To determine whether HHcy is associated with elevations in NFAT4 activity, we assessed NFAT/DNA binding in brain nuclear extracts of CT and HHcy diet mice using EMSAs. An IRDye-labeled NFAT-binding oligonucleotide probe was added to extracts to identify NFAT-DNA binding complexes. To identify NFAT isoform specific complexes, extracts were separately pretreated with monoclonal antibodies to NFATs1–4. Similar to our previous work (Furman et al., 2016), addition of NFAT4 antibody blocked the formation of a high molecular weight complex (Extended Data Fig. 3-1), which was then quantified in subsequent assays to assess NFAT4-DNA binding activity (Fig. 3*C*, arrow). Nuclear extracts from HHcy diet mice, exhibited a significant (*p* = 0.009) 35–40% increase in NFAT4-DNA binding, relative to CT diet mice (Fig. 3*D*).

10.1523/JNEUROSCI.1333-22.2023.f3-1 Figure 3-1 Using EMSA to identify NFAT4-DNA complexes in mouse brain tissue. Representative EMSA for brain homogenates from a control diet mouse. NFAT-binding probes were added to homogenates with and without antibodies (Ab) to each of the four CN-dependent NFAT isoforms (1–4) to show supershifts and/or block shifts. Unlabeled WT (wt) and mutant (mt) DNA probe was included in some conditions to demonstrate DNA-binding specificity of the labeled probe. The arrow indicates a clear block shift where the NFAT4 antibody was included with the DNA-binding probe. Download Figure 3-1, TIF file.

10.1523/JNEUROSCI.1333-22.2023.f3-2 Figure 3-2 Using EGFP expression as an indicator of GFAP promoter activity. ***A***, FOV in barrel cortex of a mouse injected with AAV-Gfa2-EGFP. Scale bar, 40 µm. Two-photon microscopy was used to visualize EGFP-expressing astrocytes (green) and cerebral vessels (red, rhodamine-dextran). Images were taken from the same FOV of the same mouse at prediet and then 1, 2, and 3 months after the initiation of HHcy diet. Scale bar, 40 µm. ***B***, High-magnification two-photon images of the hatched boxes in ***A***. Download Figure 3-2, TIF file.

Previous work on human AD brain tissue and rodent models of AD-like pathology suggest that elevated NFAT4 signaling is attributable to increased CN levels, changes in the subcellular localization of CN, and/or to the pathologic proteolysis of CN into high activity fragments (Abdul et al., 2009; Wu et al., 2010; Furman et al., 2016; Pleiss et al., 2016; Sompol et al., 2017). To determine whether HHcy treatment leads to changes in CN expression or proteolysis, we assessed CN levels in cytosolic and nuclear extracts using Western blot (Fig. 3*E*,*F*) in combination with an N-terminal CN antibody that labels both full-length CN (FL CN) and proteolized CN. Unlike rodent models of amyloid pathology, CN existed almost entirely as a 60 kDa band, indicative of FL CN (Fig. 3*E*, FL CN). Little-to-no CN proteolysis (to 57, 48, or 45 kDa fragments) was observed in either diet condition. Full-length CN was found at higher levels in cytosolic versus nuclear fractions, but no effects of diet treatment on CN levels were observed within each fraction. Together, these results suggest that increased NFAT4 activity in HHcy mice is more likely because of elevations in Ca^2+^-dependent CN activity, rather than activity arising from abnormal proteolytic processing of CN found in other disease models.

Signs of elevated CN/NFAT activity are frequently associated with reactive astrocyte phenotypes (Sompol and Norris, 2018). While the most common marker of astrocyte reactivity is elevated GFAP expression, earlier work on HHcy diet mice showed only minor changes in GFAP protein levels, despite clear changes in other astrocyte properties (Sudduth et al., 2017). Similar to earlier work, we also observed little-to-no effect of HHcy diet on brain GFAP levels using Western blot (data not shown). We therefore turned to a GFAP promoter activity assay (based on EGFP expression), which can show greater sensitivity to fluctuating GFAP levels than the GFAP protein itself (Cho et al., 2009). Mice received injections of AAV-Gfa2-EGFP vectors (Gfa2 is a full-length human GFAP promoter) into barrel cortex and were fitted with a glass cranial window. Mice were then fed with control diet or HHcy diet for 3 months, and two-photon imaging was used to assess GFAP-promoter-dependent EGFP expression. As shown in Extended Data Figure 3-2, treatment of mice with HHcy diet for 3 months results in progressively greater EGFP expression. For some mice, EGFP was fused to the VIVIT peptide (VIVIT-EGFP) to inhibit NFAT activity in astrocytes (Furman et al., 2012, 2016; Sompol et al., 2017). Figure 3, *G* and *H*, shows Gfa2-dependent EGFP expression at the prediet baseline and after 3 months of CT or HHcy diet treatment. In the prediet baseline (pre), EGFP expression was similar across groups, and there were no effects of VIVIT treatment (Fig. 3*H*). However, at the 3 month diet treatment time point, we observed a diet by VIVIT interaction (*F*_(7,89)_ = 2.873, *p* = 0.0094). EGFP volume remained relatively stable in CT diet mice, regardless of VIVIT treatment. In contrast, mice treated with HHcy diet exhibited significantly elevated EGFP levels at the 3 month time point relative to the prediet baseline (*p* = 0.008). Moreover, in HHcy mice treated with VIVIT, we observed no significant increase in EGFP levels from the prediet baseline to the 3 month time point (Fig. 3*H*). Thus, consistent with earlier studies on acute injury and amyloid rodent models, the present results suggest that CN/NFAT activity is elevated in the HHcy mouse model of VCID and linked to signs of astrocyte reactivity.

### Effects of HHcy and astrocytic CN/NFAT inhibition on cerebrovascular properties

HHcy is associated with vascular inflammation and other forms of small cerebral vessel pathology (Price et al., 2018) commonly associated with increased vessel permeability or leakiness. Immunohistochemical labeling of the basement membrane constituent collagen IV revealed no gross small vessel pathology in HHcy diet mice (Extended Data Fig. 4-1*A*,*B*). Cerebrovessel density and average vessel diameter were similar in CT and HHcy diet mice, and no effect of AAV treatment was observed (Extended Data Fig. 4-1*C*,*D*). Size distributions of cerebrovessels were also assessed with frequency histograms (Extended Data Fig. 4-1*E*). Although the distribution amplitude was slightly higher in CT diet-EGFP mice relative to all other diet-AAV treatment groups, no significant differences in major distribution properties were observed.

**Figure 4.**
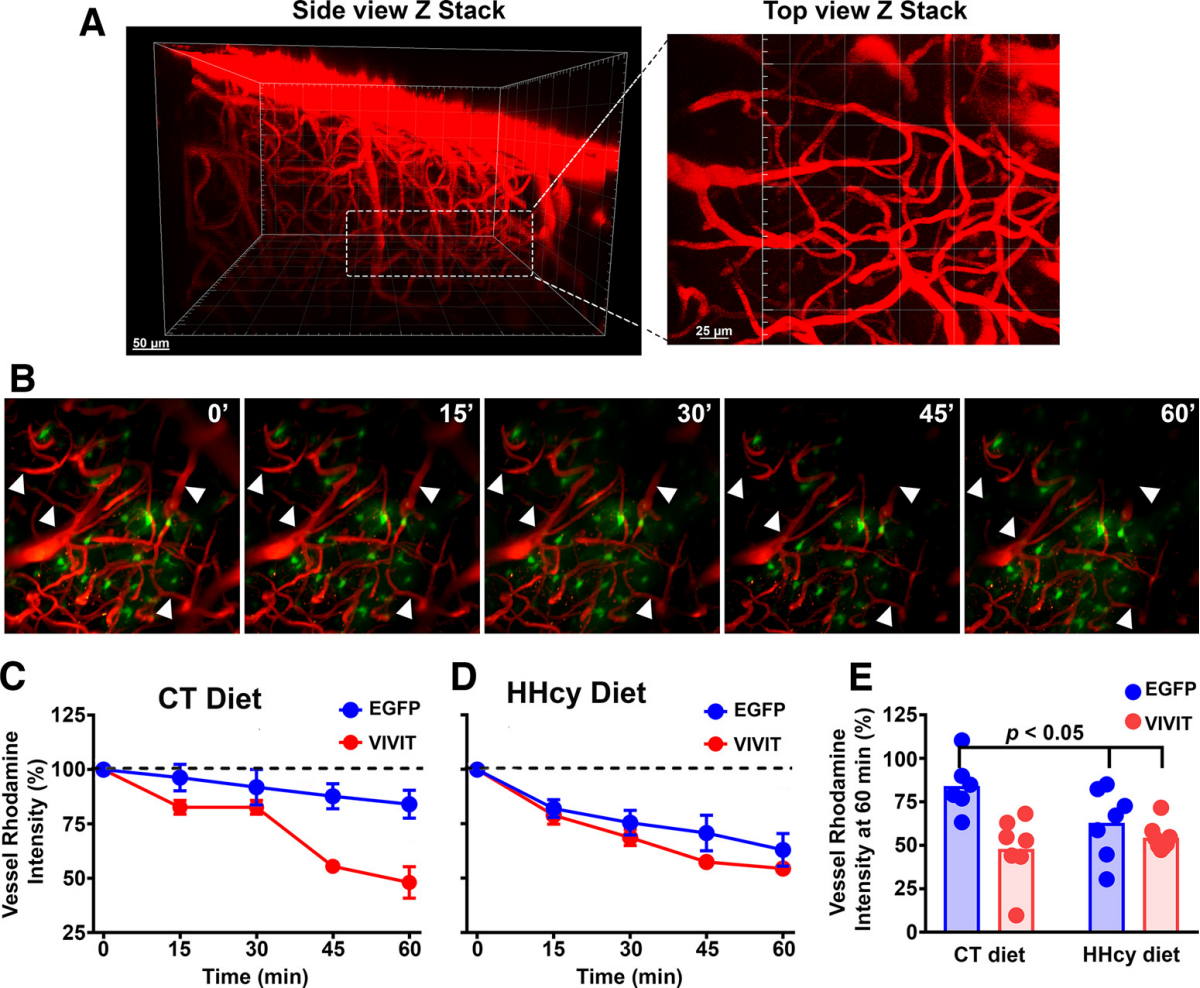
Effects of diet on cerebrovessel leakiness and impact of astrocytic NFAT inhibition. Immunohistochemical (IHC) analyses of cerebrovessels showed little difference in terms of density or average size (Extended Data Fig. 4-1), so we next functionally assessed cerebrovessels for leakiness. ***A***, Z stacks (side and top views) of two-photon images of rhodamine dextran filled cerebrovessels in mouse barrel cortex. Left, The hatched box shows where measures of cerebral vessel leakiness were acquired. ***B***, Two-photon micrographs of z-stack images acquired over a 1 h imaging session. Arrowheads point to select cerebrovessels where rhodamine fluorescence intensity was clearly lost from the 0 to the 60 min time point. ***C***, ***D***, Time plots showing the mean ± SEM vessel rhodamine fluorescence intensity across 60 min imaging sessions for CT diet (***C***) and HHcy diet mice (***D***) treated with AAV-Gfa2-EGFP (blue) or AAV-Gfa2-VIVIT (red). Values are expressed as percentage of the 0 min time point. ***E***, Scatter plots showing vessel rhodamine fluorescence intensity at the 60 min time point (percentage of 0 min time point) in diet and AAV treatment groups. Greater loss of rhodamine fluorescence was associated with HHcy versus CT diet. Interestingly, VIVIT also led to greater loss of rhodamine fluorescence in the CT diet group but not the HHcy diet group. Each data point represents an individual mouse (*n* = 6–7 mice/group). Significance determined with rmANOVA followed by paired *t* tests (pre vs 3 months), within diet group.

To estimate vessel leakiness in CT and HHcy diet mice, two-photon imaging was used to quantify the extent of rhodamine-dextran (40 kDa) dissipation from cerebral vessels in barrel cortex (Fig. 4*A*,*B*). The impact of astrocyte signaling was also addressed with the use of AAV-Gfa2 vectors expressing EGFP control or VIVIT-EGFP to inhibit NFAT activation. Vessels were imaged to a depth of 200–300 µm once every 15 min across a 60 min period (Fig. 4*B*), and rhodamine labeling intensity in cerebrovessels was calculated as a ratio to local EGFP expression in astrocytes. As shown in Figure 4, *C* and *D*, all diet and AAV treatment groups exhibited a significant loss of rhodamine intensity over time, suggestive of vessel leakiness. However, the rate of rhodamine leakage was significantly affected by an interaction between diet and AAV (*F*_(1,24)_ = 5.0, *p* = 0.03). *Post hoc* analyses showed that VIVIT treatment led to faster and more extensive leakage in the CT diet group (Fig. 4*C*,*E*; *p* < 0.05), but not in the HHcy diet group (Fig. 4*D*,*E*). HHcy diet-EGFP-treated mice also exhibited faster and more extensive leakage relative to the CT diet-EGFP group (Fig. 4*E*; *p* < 0.05). In contrast to vessel lumen analyses, we observed little to no changes in extravascular rhodamine fluorescence intensity levels (data not shown), regardless of diet of AAV treatment. These results suggest that VIVIT and HHcy diet promote cerebrovessel leakiness, but there is no additive effect of HHcy and VIVIT.

10.1523/JNEUROSCI.1333-22.2023.f4-1 Figure 4-1 Microvessel labeling in diet/AAV-treated mice. ***A***, ***B***, Mice received intrahippocampal injections of AAV-Gfa2-EGFP or AAV-Gfa2-VIVIT. At 1 month after AAV injections, mice were fed for an additional 3 months with CT diet or HHcy diet. Formalin-fixed brain sections were then prepared and labeled for the basement membrane constituent, collagen IV, to reveal microvessels in the hippocampus (***B***). ***C***, ***D***, Neither the number of labeled vessels per mm^2^ (***C***), nor the average vessel diameter (***D***) was affected by diet or AAV treatment. ***E***, Frequency histograms showing the distribution of microvessel diameters across diet-AAV treatment conditions. Although the distribution showed a higher amplitude in the CT diet-EGFP group, other histogram parameters were similar, and no significant differences were observed across groups using either chi-square tests or Kolmogrov–Smirnov tests. Download Figure 4-1, TIF file.

**Figure 5.**
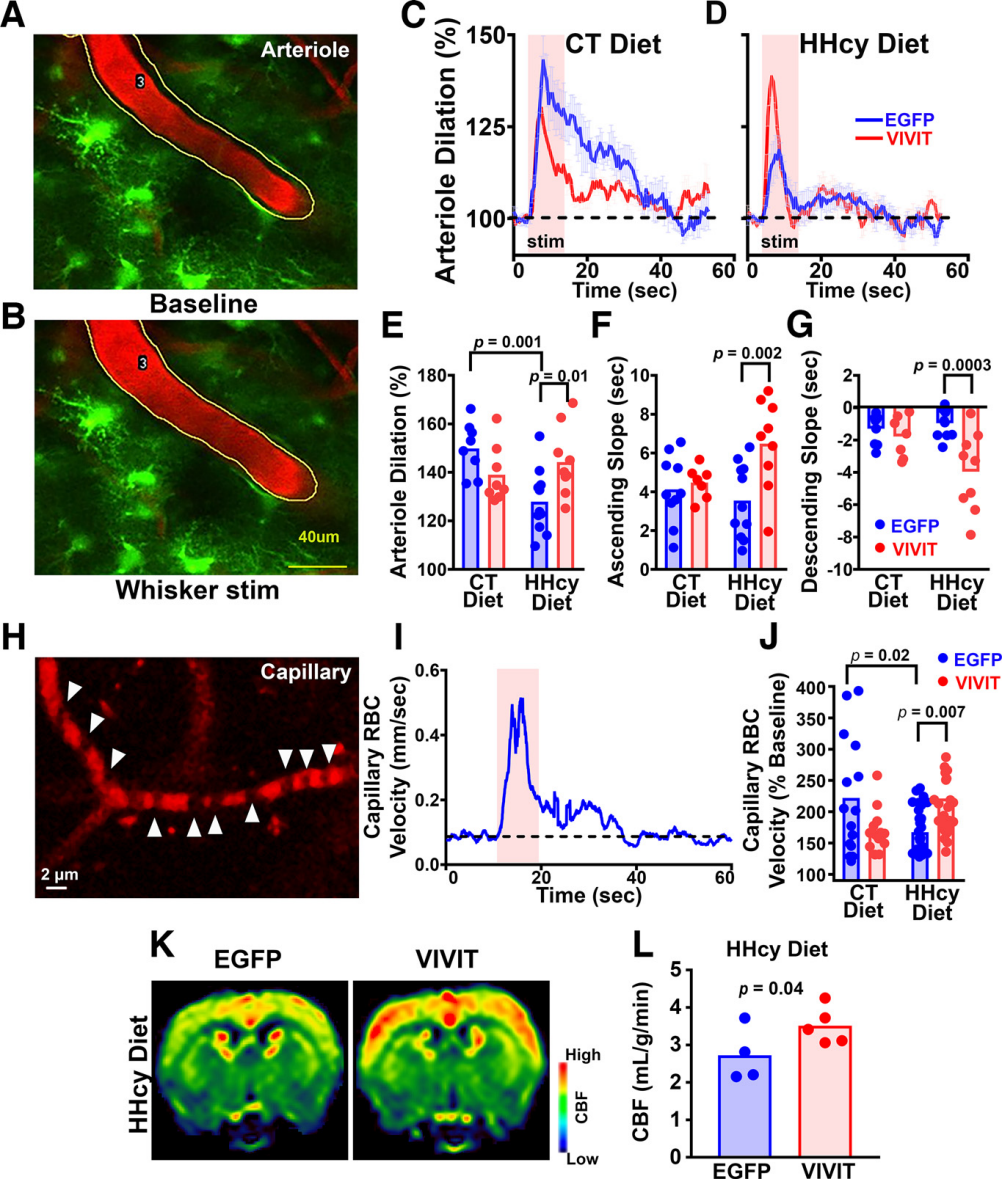
Effects of diet on neurovascular coupling and impact of astrocytic NFAT inhibition. ***A***, ***B***, Representative two-photon images of penetrating arterioles (red) in a fully awake mouse fed with CT diet and pretreated with AAV-Gfa2-EGFP control vectors (EGFP-expressing astrocytes are green). Images were taken before ***A*** (baseline) and during ***B*** (whisker stimulation) air-puff stimulation of the contralateral vibrissae (10 Hz, 10 s). The hatched lines in the baseline image (***A***) mark the perimeter of the dilated vessel shown during stimulation (***B***). Scale bar, 40 µm. ***C***, ***D***, Time plots showing maximum arteriole volume (mean ± SEM) measured before, during, and after whisker stimulation (pink rectangle) in CT diet (***C***) and HHcy diet (***D***) mice treated with AAV-Gfa2-EGFP (blue) or AAV-Gfa2-VIVIT (red). Values are expressed as a percentage of the prestimulation baseline. ***E–G***, Scatter plots showing maximal arteriole dilation (***E***), dilation onset (***F***), and dilation offset (***G***) during whisker stimulation as a function of diet and AAV treatment; *n* = 8–11 mice/group. Relative to CT diet mice, dilatory responses were reduced in amplitude/magnitude (***E***) in HHcy mice treated with control AAV-Gfa2-EGFP. Conversely, treatment of HHCy mice with AAV-Gfa2-VIVIT prevented these deficits. VIVIT also accelerated the onset/offset kinetics of arteriole dilation in HHcy mice (***F***, ***G***). Each data point represents an individual mouse (*n* = number of mice/group). ***H***, Two-photon micrograph of a barrel cortex capillary. Line scans along the horizontal axis (yellow) line were used to track the movement of RBCs (dark spots along the capillary, white arrowheads) through the capillary lumen. ***I***, Representative time plot showing the change in maximum RBC velocity during air-puff whisker stimulation (pink rectangle). ***J***, Scatter plot showing maximum capillary RBC velocity (normalized to baseline) across diet and AAV treatment groups; *n* = 13–31 capillaries/group. Air-puff-mediated elevations in RBC velocity were reduced in HHcy EGFP mice relative to the CT diet-EGFP and HHcy-VIVIT groups. ***K***, Representative pseudo-colored images showing CBF in HHcy mice treated with AAV-Gfa2-EGFP (*n* = 4) or AAV-Gfa2-VIVIT (*n* = 5) as measured with pCASL MRI. The CBF level is colorized in a linear scale (far right). ***L***, Scatter plot showing quantified CBF levels in EGFP- and VIVIT-treated HHcy diet mice. CBF was elevated in the VIVIT group. Significance for ***A–J*** determined with two-way ANOVA and Sidak's multiple-comparisons tests. Significance for ***L*** and ***K*** determined with unpaired *t* test.

To evaluate cerebrovascular function, we next assessed vasodilatory responses in penetrating arterioles before and during whisker stimulation. The role of astrocytes in neurovascular coupling remains controversial (Rosenegger and Gordon, 2015; Filosa et al., 2016; Kisler et al., 2017), and relatively few studies have specifically investigated how cerebrovascular function is affected by astrocytes in the context of pathologies relevant to AD and/or ADRDs (Tarantini et al., 2017; Stackhouse and Mishra, 2021). Mice received injections of AAV-Gfa2-EGFP or AAV-Gfa2-VIVIT into barrel cortex and were then fed with control or HHcy diet for 3 months. Penetrating arterioles, chosen randomly, were identified at a depth within 150 µm in active barrel regions, and several vessel parameters, including average vessel volume and dilation rise and decay times, were measured before and during a 10 s air-puff train to the contralateral whiskers (Fig. 5*A*,*B*). Time plots in Figure 5, *C* and *D*, show normalized arteriole volume measures before, during, and after whisker stimulation for AAV-treated CT diet mice (Fig. 5*C*) and HHcy diet mice (Fig. 5*D*), respectively. All groups showed significant arteriole dilation during whisker stimulation, but there were clear deficits in the HHcy-EGFP treatment group (Fig. 5*D*). Differences across treatment groups were further evaluated with two-way ANOVA (Fig. 5*E*), which revealed a significant diet by AAV interaction for maximum dilation (*F*_(1,32)_ = 10.42, *p* = 0.003). Sidak's *post hoc* analyses of simple main effects showed that HHcy diet-EGFP mice exhibited a significant deficit relative to CT diet-EGFP mice (*p* = 0.001), whereas treatment of HHcy diet mice with AAV-Gfa2-VIVIT significantly increased maximal vasodilation (HHcy-EGFP vs HHcy-VIVIT, *p* = 0.01). The latency to maximal dilation during whisker stimulation (Fig. 5*F*, ascending slope) and the following return to baseline vascular tone (Fig. 5*G*, descending slope) both exhibited significant diet by AAV interactions (ascending, *F*_(1,33)_ = 4.495, *p* = 0.04; descending, *F*_(1,31)_ = 5.996, *p* = 0.02). Treatment of HHcy diet mice with AAV-Gfa2-VIVIT resulted in faster onset and offset of dilatory responses compared with their EGFP-treated counterparts (ascending, *p* = 0.002; descending, *p* = 0.0003). Neither the onset nor offset of arteriole dilation were specifically affected by diet.

Blood flow through cerebral capillaries is a major component of overall brain perfusion (Hudetz, 1997; Jespersen and Østergaard, 2012; Grubb et al., 2020; Hartmann et al., 2021) and a likely contributor to impaired cerebrovascular function and cognitive decline in AD and ADRDs (Gutiérrez-Jiménez et al., 2018; Cruz Hernández et al., 2019; Ali et al., 2022). Thus, in addition to assessing dilatory responses in penetrating arterioles, we also measured RBC velocity in multiple barrel cortex capillaries from each mouse (Fig. 5*H*,*I*; ∼83 capillaries across four groups). Line scans were taken along the horizontal axis of capillaries before, during, and after whisker stimulation (Fig. 5*H*), and the maximum increase in RBC velocity was assessed relative to the pre-air-puff baseline period (Fig. 5*I*). We attempted to measure from three capillaries per mouse, but movement artifacts prevented the collection of interpretable measures from all mice. The numbers shown in Figure 5*J* (left) therefore represent the number of capillaries assessed in each diet/treatment group (i.e., from 6 CT EGFP, 5 CT VIVIT, 11 HHcy EGFP, and 9 HHcy VIVIT mice, respectively). All groups exhibited a significant stimulation-dependent increase in RBC velocity, and a two-way ANOVA indicated that neither diet nor AAV treatment significantly affected the time to reach maximum velocity (data not shown). However, there was a significant diet by AAV interaction for the maximum velocity achieved (*F*_(1,79)_ = 11.9, *p* = 0.0009). The stimulus-induced increase in RBC velocity was significantly reduced by HHcy diet (CT diet-EGFP vs HHcy diet-EGFP, *p* = 0.02) and this deficit was significantly reduced by treatment with AAV-Gfa2-VIVIT (HHcy EGFP vs HHcy VIVIT, *p* = 0.007). Together with the arteriole volume measures, these results demonstrate that HHcy diet impairs neurovascular coupling and that inhibition of astrocytic NFAT signaling reduces this deficit.

Previous work from our research groups showed that HHcy diet led to reduced CBF in mice, as measured with pCASL MRI (Braun et al., 2019). A similar approach was used here to determine whether inhibition of astrocytic NFAT signaling improves CBF in a small cohort of HHcy diet mice. Mice received bilateral intrahippocampal injections of AAV-Gfa2-EGFP (*n* = 4) or AAV-Gfa2-VIVIT-EGFP (*n* = 5) and were started on HHcy diet 1 month later. After 14 weeks on diet, mice were anesthetized with 4% isoflurane, and pCASL MRI was used to assess perfusion in bilateral hippocampus. Figure 5*K* shows the heat map with CBF level colorized in a linear scale (right), and the corresponding CBF values in the bilateral hippocampus are shown in Figure 5*L*. The results showed that CBF (ml/g/min) was significantly greater in VIVIT versus EGFP-treated mice (Fig. 5*L*; *p* = 0.04). The results suggest that inhibition of astrocytic NFAT signaling alleviates cerebral hypoperfusion deficits, in addition to improving functional hyperemia in HHcy mice.

### Inhibition of astrocytic CN/NFAT activity improves synaptic function and cognition in HHcy diet mice

**Figure 6.**
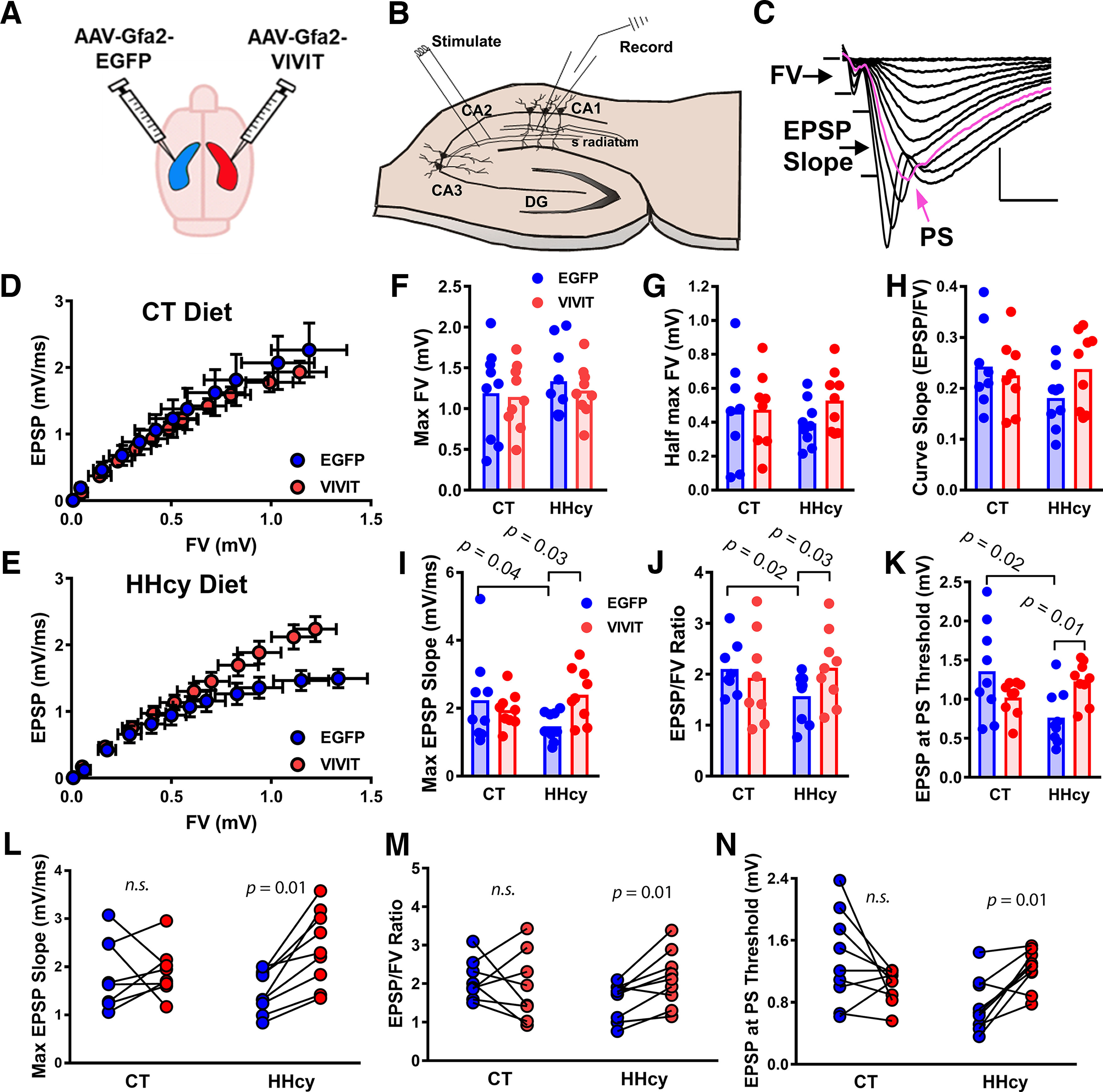
Effects of diet on basal synaptic strength and impact of astrocytic NFAT inhibition. ***A***, Mice treated with CT or HHcy diet each received an intrahippocampal injection of AAV-Gfa2-EGFP (control AAV) into one hemisphere (blue) and AAV-Gfa2-VIVIT into the other hemisphere (red). ***B***, At the end of diet treatment, coronal sections were prepared, and EPSPs were recorded from CA1 stratum radiatum after stimulation of CA3 Schaffer collaterals. ***C***, Representative EPSP waveforms at increasing stimulus intensities along with the primary parameters investigated, that is, FV, EPSP slope, and PS threshold (shown in pink). ***D***, ***E***, Mean ± SEM EPSP slopes plotted against corresponding FV amplitudes (mean ± SEM) across 12 increasing stimulus levels in CT diet and HHcy diet mice (blue, AAV-Gfa2-EGFP hemisphere; red, AAV-Gfa2-VIVIT hemisphere). ***F*–*K***, Field potential parameters extracted from the synaptic strength curves shown in ***D***, ***E***, maximum FV amplitude (***F***), half-maximum FV (***G***), curve slope (***H***), maximum EPSP slope (***I***), EPSP/FV ratio (***J***), and PS threshold (***K***). ***L–N***, Interhemispheric comparisons within each mouse illustrating AAV-treatment effects on the maximal EPSP slope (***L***), the EPSP/FV ratio (***M***), and the PS threshold in mice fed with CT or HHcy diet. Field potential parameters in HHcy mice were consistently improved by treatment with AAV-Gfa2-VIVIT. Each data point represents field potential values averaged within each hemisphere per mouse (*n* = 8–9 mice per diet group). Diet effects were detected with two-way ANOVA. Within-animal AAV effects were determined with paired *t* tests. n.s. non-significant.

Previously, we showed that reactive astrocyte signaling via the CN/NFAT pathway led to synaptic dysfunction in rodent models of injury and AD-like pathology (Furman et al., 2012, 2016; Sompol et al., 2017). To determine whether similar astrocyte-dependent deficits are present in the HHcy model of VCID, we injected hippocampi of diet-fed mice with AAV vectors and used electrophysiologic approaches to assess multiple CA3–CA1 signaling properties in *in situ* brain slices (Fig. 6*A–C*). Each mouse received an intrahippocampal injection of AAV-Gfa2-EGFP control vector in one hemisphere and an intrahippocampal injection of AAV-Gfa2-VIVIT into the other hemisphere so that each mouse served as its own internal control (Fig. 6*A*). CA3 axons were stimulated with a bipolar electrode, and evoked responses were collected extracellularly via a recording electrode in CA1 stratum radiatum (Fig. 6*B*). Field potentials were recorded across increasing stimulus intensities to extract presynaptic FV amplitudes, corresponding EPSP slopes, and population spikes (PSs; Fig. 6*C*). Within the CT diet group, AAV treatment had little effect on synaptic strength curves across hemispheres (Fig. 6*D*). Conversely, synaptic strength curves for AAV-Gfa2-VIVIT-treated hemispheres in HHcy mice exhibited an upward and leftward shift (Fig. 6*E*), suggesting that VIVIT improved synaptic strength in HHcy diet mice. Multiple parameters were extracted from EPSP/FV curves and compared across diet/AAV treatment conditions including maximal and half-maximal FV (Fig. 6*F*,*G*), curve slopes (Fig. 6*H*), maximal EPSP slope, and EPSP/FV ratio (Fig. 6*I*,*J*), and PS threshold (i.e., EPSP slope associated with the first appearance of a PS in the ascending limp of the field potential; Fig. 6*K*). Maximal FV amplitudes, half-maximal input, and curve slopes, which reflect the overall level of presynaptic input into the CA1 region, were not significantly affected by either diet or AAV treatment, although half-maximal input and curve slope were modestly reduced in the EGFP-treated hemisphere of HHcy diet mice (Fig. 6*G*,*H*). Conversely, a significant diet by AAV treatment interaction was observed for maximal EPSP slope (*F*_(1,16)_ = 7.31, *p* = 0.01) and EPSP/FV ratio (*F*_(1,16)_ = 4.35, *p* = 0.05), characterized by deficits in the EGFP-treated, but not in the VIVIT-treated, hemisphere of HHcy mice (Fig. 6*I*,*J*). A similar interaction was also revealed for PS threshold (*F*_(1,16)_ = 11.93, *p* = 0.003), characterized by a reduced threshold in the EGFP-treated, but not in the VIVIT-treated, hemisphere of HHcy mice (Fig. 6*K*). Follow-up interhemispheric comparisons of postsynaptic parameters within each mouse further highlights and confirms that VIVIT treatment improved synapse function and reduced excitability in HHcy-treated, but not in CT diet–treated mice (Fig. 6*L–N*).

**Figure 7.**
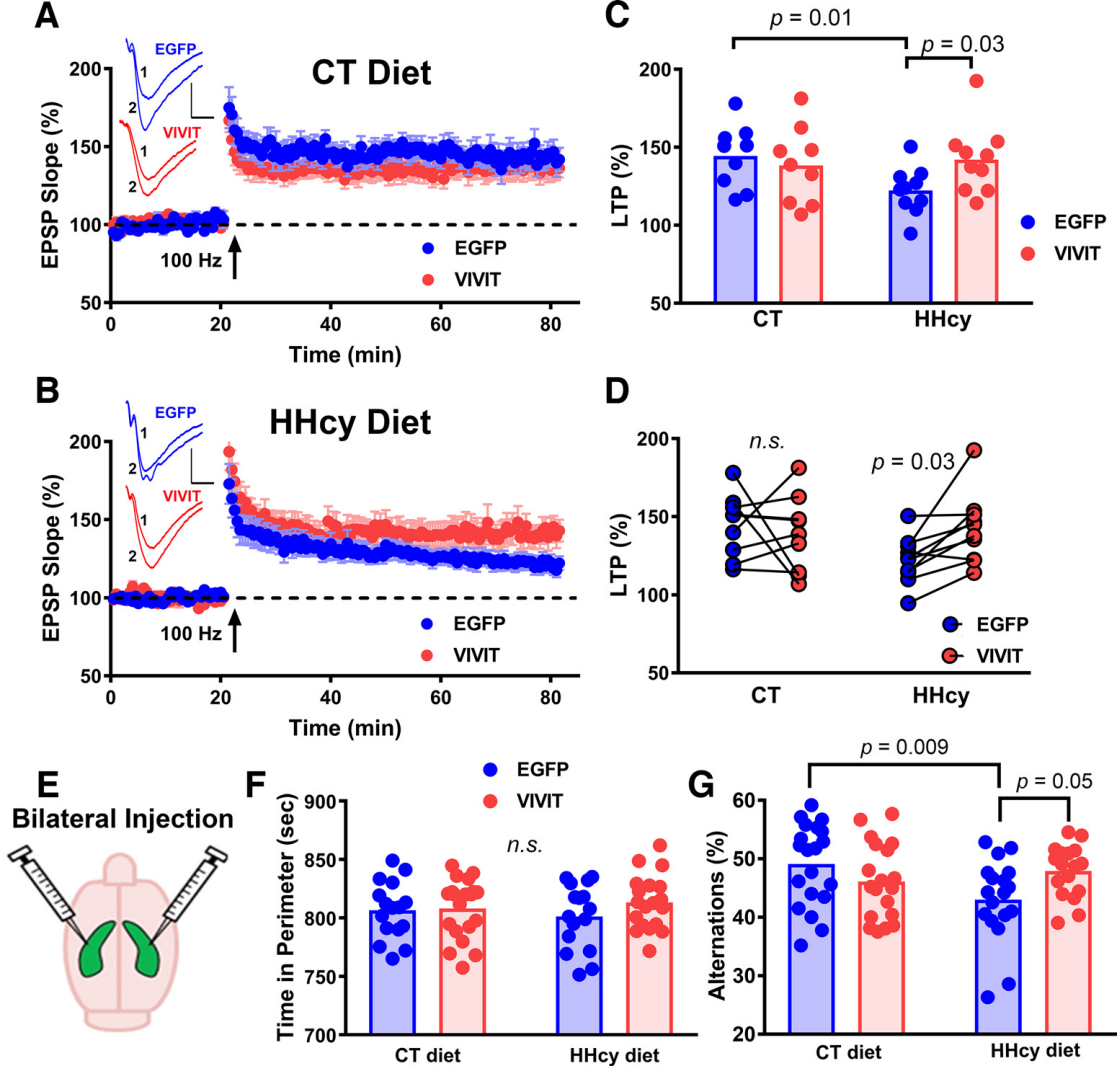
Effects of diet on hippocampal-dependent synaptic plasticity and behavior and the impact of astrocytic NFAT inhibition. ***A–D***, CT diet and HHcy diet mice that received intrahippocampal injections of AAV-Gfa2-EGFP (control AAAV) into one hemisphere and AAV-Gfa2-VIVIT into the other hemisphere (same mice from Fig. 5). Time plots showing mean ± SEM EPSP slopes (normalized to baseline) in CT diet mice (***A***) or in HHcy diet mice (***B***) collected before and after the delivery of two 100 Hz stimulus trains (1 s duration/separated by 10 s). Blue plot symbols are from recordings collected in the AAV-Gfa2-EGFP hemisphere, and red plot symbols show recordings collected from the AAV-Gfa2-VIVIT hemisphere. Insets, Representative averaged EPSP waveforms from each AAV-treated hemisphere before (1) and after (2) 100 Hz stimulation. Calibration: 1 mV, 5 ms. ***C***, Scatter plots showing average LTP amplitude in each AAV/diet treatment group. ***D***, Interhemispheric comparisons of LTP levels within each mouse treated with CT or HHcy diet. LTP was generally reduced in the EGFP-treated (control AAV) hemisphere of HHcy diet mice. This HHcy-mediated deficit was prevented by astrocytic NFAT inhibition with VIVIT. Each data point represents field potential values averaged within each hemisphere per mouse (*n* = 8–9 mice per diet group). Diet effects were detected with two-way ANOVA. Within-animal AAV effects were determined with paired *t* tests. ***E***, For behavioral assessments, CT diet and HHcy diet mice received bilateral hippocampal injections of either AAV-Gfa2-EGFP or AAV-Gfa2-VIVIT. ***F***, Mean time spent near the perimeter of an open field maze. ***G***, Mean alternations (%) on a Y maze. HHcy-related deficits on the Y maze were prevented by treatment with AAV-Gfa2-VIVIT. Each data point in ***F*** and ***G*** represent an individual mouse; *n* = 17–20 mice per group. Significance determined for behavioral assays using two-way ANOVA followed by Sidak's multiple-comparisons tests. n.s. non-significant.

After completion of synaptic strength curves, stimulation intensity in each slice was reset to elicit an ∼1 mV EPSP. A 20 min baseline was then collected followed by delivery of two 100 Hz trains (10 s intertrain interval) to induce LTP. Potentiated responses were collected across an additional 60 min (Fig. 7*A*,*B*). Similar to input-output analyses (Fig. 6), a two-way rmANOVA detected a significant diet by AAV interaction (*F*_(1,16)_ = 4.46, *p* = 0.04), indicative of reduced LTP in HHcy mice, but only in the EGFP-treated hemisphere (Fig. 7*B*,*C*). Follow-up interhemispheric comparisons of LTP levels within each mouse further highlights and confirms that VIVIT treatment protected or enhanced LTP in HHcy-treated, but not in CT diet–treated mice (Fig. 7*D*).

Finally, effects of inhibiting astrocytic CN/NFAT signaling were assessed on HHcy-related cognitive deficits. Mice received bilateral intrahippocampal injections of AAV-Gfa2-EGFP control or AAV-Gfa2-VIVIT vectors (Fig. 7*E*) and were then fed for 12 weeks with CT diet or HHcy diet and tested on an open field maze to assess anxiety (Fig. 7*F*) and a Y maze (Fig. 7*G*) to assess hippocampal-dependent spontaneous alternation behavior. No diet or AAV effects were observed for open field maze performance (Fig. 7*F*). However, a two-way ANOVA on Y maze performance revealed a significant diet by AAV interaction (*F*_(1,70)_ = 7.017, *p* = 0.01; Fig. 7*G*). Sidak's multiple-comparisons tests showed that alternations were reduced in HHcy-EGFP mice compared with CT-EGFP mice (*p* = 0.009). However, HHcy mice treated with AAV-Gfa2-VIVIT performed similarly to CT mice and exhibited superior performance compared with HHcy mice treated with AAV-Gfa2-EGFP control vector (*p* = 0.05). Together, the results show that HHcy diet disrupts hippocampal synaptic function and hippocampal-dependent cognition through a mechanism that involves reactive astrocyte signaling.

## Discussion

Using a diet-based model of VCID, we show that perturbed astrocyte signaling negatively affects key functional components of the neurovascular unit—neurovascular coupling and synaptic transmission/plasticity. The results provide the first evidence that HHcy leads to astrocytic Ca^2+^ dysregulation and elevated astrocytic CN/NFAT4 signaling. These changes were linked to both neurovascular coupling deficits and synaptic impairments, as demonstrated by the mostly protective outcomes observed following treatment with AAV-Gfa2-VIVIT vectors to suppress astrocytic CN/NFAT activity.

### Astrocyte Ca^2+^ dysregulation in the HHcy diet model

Ca^2+^ signaling and neurovascular coupling (NVC) responses were investigated in fully awake mice to avoid potentially confounding effects of anesthesia on vascular tone and cellular activity (Toda et al., 2007; Franceschini et al., 2010; Gao et al., 2017). When in the fully awake state, engagement of the barrel cortex via whisker stimulation elicited rapid Ca^2+^ signals in astrocytes that were highly coordinated across cells within the field of view. Similar to a previous report on awake mice (Tran et al., 2018), we found that Ca^2+^ elevations in astrocytes, even in astrocyte end feet, occurred after arteriole dilatory responses, suggesting that astrocytic Ca^2+^ transients are unlikely to trigger vasodilation, at least under the whisker-puff conditions used here. Interestingly, treatment of mice with HHcy diet was associated with impaired functional connectivity within astrocyte networks. Specifically, fewer astrocytes showed Ca^2+^ activity during whisker stimulation, and fewer showed coordinated activity with one another. Previous reports have shown that Ca^2+^ signaling in astrocytes is driven to a great extent by direct neuronal activity and neurotransmitter release (Perea and Araque, 2005; Wang et al., 2006; Tran et al., 2018) and also by signals originating from the blood or the blood vessels (Kim et al., 2016). The dysfunction of synapses and the impairment of cerebrovascular regulation arising from HHcy diet treatment, as shown in Figures 5–7 and in earlier work, could each contribute to the diminished activation of astrocytes, either by acting alone or in concert. Alternatively, HHcy is known to cause perivascular inflammation and the release of cytokines that have been shown to trigger dysfunctional changes in connexins and/or gap junction coupling (Même et al., 2006; Kielian, 2008), which could limit signaling across the astrocyte syncytium. Regardless, it is presently unknown if altered Ca^2+^ signaling across astrocyte networks in HHcy mice is a cause or an effect of other pathologic and functional changes arising in this model.

In contrast to diminished network signaling properties, those astrocytes that did respond to whisker stimulation exhibited significantly greater Ca^2+^ transient magnitudes. These observations suggest that individual astrocytes in HHcy mice either respond more robustly to cortical activation in an effort to compensate for impaired network activity, and/or they show dysfunctional Ca^2+^ handling. Dysregulation of Ca^2+^ is a hallmark of both neurons and astrocytes in animal models of aging and disease (Frazier et al., 2017; Verkhratsky et al., 2017; Sompol and Norris, 2018; Lim et al., 2021). Several studies have reported astrocytic Ca^2+^ dysregulation in rodents with amyloid or vascular pathology indicated by larger Ca^2+^ transients or more frequent Ca^2+^ oscillations (Winship and Murphy, 2008; Ding et al., 2009; Kuchibhotla et al., 2009; Delekate et al., 2014). Although augmented Ca^2+^ responses are unlikely to be a proximal cause of impaired arteriolar responses in HHcy mice (because Ca^2+^ transients arise after vasodilation; Fig. 1*N*), elevated Ca^2+^ signaling could be an upstream cause of CN/NFAT hyperactivity and subsequent astrocyte reactivity. Although also found in pericytes (Filosa et al., 2007; Blanchard et al., 2020), NFAT4 is most prominently expressed in astrocytes (especially relative to neurons and microglia; Fig. 3*A*) and tends to exhibit elevated activity in astrocytes that are reactive. In a rat model of traumatic brain injury (TBI) and in the 5xFAD mouse model of amyloid pathology, NFAT4 activity was 2.5–4-fold higher than in wild-type mice and sham-injured rats, respectively, and was associated with irreversible proteolytic modifications to CN resulting in constitutive Ca^2+^-independent phosphatase activity (Furman et al., 2016; Sompol et al., 2017). However, unlike amyloidogenic mice and TBI rats, there was no evidence for changes in CN expression or proteolysis in HHcy mice (Fig. 3*E*,*F*). Thus, the comparably smaller increase in NFAT signaling (35–40%) found with HHcy treatment was most likely attributable to elevated Ca^2+^ levels leading to increased Ca^2+^/calmodulin-dependent CN activity (rather than constitutive Ca^2+^-independent activity).

It is presently unknown what mechanisms or Ca^2+^ sources contribute to astrocytic Ca^2+^ dysregulation and CN/NFAT4 hyperactivation because of HHcy. Previous work implicated P2Y1 receptor activation as the mechanism for augmented Ca^2+^ transient oscillations in APP/PS1 (amyloid precursor protein and presenilin-1) transgenic mice (Delekate et al., 2014), and P2Y1 receptor activation has been shown to stimulate CN/NFAT activity in primary astrocytes (Pérez-Ortiz et al., 2008). Interestingly, the P2Y1 receptor was among several Ca^2+^ signaling pathway constituents that was upregulated by elevated CN expression/activity (Norris et al., 2005). CN has also been shown to directly stimulate elevated Ca^2+^ transients in astrocytes in response to Aβ peptides (Lim et al., 2013). Furthermore, it has been shown that homocysteine is an agonist at the glutamate binding site and partial antagonist of the glycine coagonist sites of neuronal NMDA receptors, where it may drive excessive Ca^2+^ influx and neurotoxicity (Lipton et al., 1997). The possibility that homocysteine modulates Ca^2+^ signaling through astrocytic NMDA receptors has yet to be investigated. Together, these observations suggest that Ca^2+^ dysregulation and CN hyperactivation may involve a deleterious feedback loop that maintains reactive astrocyte phenotypes (Sama et al., 2008; Price et al., 2021). However, further work is needed to clarify the mechanistic interactions between Ca^2+^, CN, and astrocyte reactivity in the context of AD and ADRDs.

### Protection of cerebrovascular and synaptic function through inhibition of astrocytic CN/NFAT activity

HHcy mice exhibited impairments in functional hyperemia, which is consistent with previous reports showing progressive microvascular pathology and cerebral hypoperfusion with progression on the HHcy diet (Sudduth et al., 2013; Braun et al., 2019). The results also support many other studies that have shown neurovascular coupling deficits in cognitively impaired animals as a consequence of aging, AD-like pathology, traumatic brain injury, cerebrovascular pathology, and/or metabolic dysfunction (Girouard & Iadecola, 2006; Nelson et al., 2016; Csiszar et al., 2017; Jang et al., 2017; Tarantini et al., 2017). Although most of these animal models exhibit varying degrees of astrocyte reactivity, most studies on the role of astrocytes in neurovascular coupling have used otherwise healthy adult animals where there is presumably little astrocyte reactivity. Moreover, methodology across these studies has varied wildly (e.g., *in situ* slices vs intact brain, presence of anesthesia, intensity of stimulation, specific vessel types examined, etc.), which may contribute to the overall lack of consensus in the field regarding astrocytes in vasomodulation and other cerebral vessel properties. The present study differed from most of the previous work in that we assessed cerebral vessel function in fully awake rodents and targeted signaling pathways involved in the regulation of astrocyte reactivity rather than specific vasomodulatory targets. Inhibition of astrocytic NFATs improved arteriole dilatory responses and red blood cell velocity in cerebral capillaries in HHcy diet mice, but did not reduce vessel leakiness. Basal cerebral perfusion in anesthetized HHcy mice was also improved by VIVIT. As far as we know, this is the first study to directly assess the impact of reactive astrocytes in general, and CN/NFAT signaling in particular, on cerebrovascular function. The results suggest that reactive astrocyte signaling is not conducive to the dynamic coupling of cerebral blood flow to neuronal energy demands.

Rhodamine-dextran fluorescence intensity was also lost in the lumen of cerebrovessels at a greater rate in HHcy diet mice. The extent and rate of change were not altered by inhibition of astrocytic CN/NFAT signaling. In fact, VIVIT delivery to astrocytes actually promoted the loss of vascular rhodamine fluorescence in CT diet mice, suggesting a possibly detrimental outcome of inhibiting CN/NFATs under otherwise healthy conditions (discussed further below). Although this finding suggests that HHcy is exacerbating blood–brain barrier (BBB) permeability, the major caveat to our analysis is that we did not observe a corresponding increase in parenchymal rhodamine dextran fluorescence as reported in other studies on aging and/or VCID mouse models (Biancardi et al., 2014; He et al., 2017; Ju et al., 2018; Lee et al., 2018; Nyúl-Tóth et al., 2021; George et al., 2022). It is possible that our imaging methods lacked the spatial and temporal sensitivity to observe robust accumulation of extravascular rhodamine, or it might suggest that structural damage to the BBB in the HHcy diet model is not as extensive as other models. These possibilities will need to be evaluated in future studies.

Similar to previous work on mice with amyloid pathology (Furman et al., 2012; Sompol et al., 2017) and traumatic brain injury (Furman et al., 2016), we found here that blockade of astrocytic CN/NFAT signaling protects synaptic function and plasticity in a mouse model of VCID. Notably, this was the first investigation of synaptic function in the HHcy diet model. And although HHcy diet caused synaptic deficits that were qualitatively similar to what we've observed before in mice with amyloid pathology (i.e., reduced synaptic strength and LTP), the deficits in HHcy mice appeared to be milder (and more variable) in extent. Nonetheless, within-mouse comparisons in the HHcy group showed that the synaptoprotective effects of AAV-Gfa2-VIVIT were remarkably consistent. Also, similar to amyloid models, HHcy mice exhibited a reduced population spike threshold, which is consistent with excitotoxic effects previously reported with hyperhomocysteinemia (Olney et al., 1987; Kim and Pae, 1996; Lipton et al., 1997; Zieminska et al., 2006; Ganapathy et al., 2011). AAV-Gfa2-VIVIT normalized the population spike threshold in HHcy mice, confirming that CN/NFAT signaling diminishes the capacity of reactive astrocytes to protect against glutamate toxicity (Sama et al., 2008; Abdul et al., 2009; Sompol et al., 2017). Because of the overall beneficial effects of AAV-Gfa2-VIVIT to synaptic function/plasticity, it is perhaps not surprising that bilateral intrahippocampal injections of AAV-Gfa2-VIVIT also improved performance on a hippocampal-dependent cognitive task. Together, the results from our previous and present work suggest that reactive astrocyte signaling is a common mechanism of neural dysfunction and cognitive decline in AD and VCID.

A major unresolved question from this work is whether improved cerebrovascular function with AAV-Gfa2-VIVIT treatment arises from the stabilization and protection of neuronal function and viability, or whether neuronal function is protected because of the beneficial effects of VIVIT on cerebral vessel function. In astrocytes, the CN/NFAT pathway exerts extensive control over transcriptional mechanisms associated with reactive phenotypes (Norris et al., 2005; Fernandez et al., 2007). Many of these mechanisms are directly related to synapse dysfunction and neuronal hyperexcitability (e.g., matricellular factors, complement factors, and glutamate transporters), whereas others are related to the cerebrovasculature (e.g., adhesive glycoproteins, matrix metalloproteinases) and vasomodulation (prostaglandins and receptors, angiotensin pathway constituents; Norris et al., 2005; Canellada et al., 2008; Serrano-Pérez et al., 2011). Still other pathways are associated with broader indicators of overall tissue health and viability (e.g., inflammatory mediators, Ca^2+^ signaling mediators/regulators, metabolic proteins/enzymes, redox state modulators; Norris et al., 2005; Fernandez et al., 2007; Canellada et al., 2008; Sama et al., 2008) that may limit the dynamic metabolic interactions between neurons and the cerebrovasculature. Clearly, further work is necessary to determine whether reactive astrocytes negatively affect synapses and blood vessels through unique mechanisms that are specialized to purpose, or through general broad-acting mechanisms that promote overall brain health.

### Differential effects of AAV-Gfa2-VIVIT in CT diet and HHcy diet mice

Finally, for several cerebrovascular and synaptic end-point measures, inhibition of astrocytic NFAT signaling with VIVIT appeared to cause possibly detrimental effects in CT diet animals. Previously, we found that AAV-Gfa2-VIVIT, or the NFAT inhibitor Q134R, was associated with microglial reactivity and/or reduced synaptic strength in healthy wild-type mice (Furman et al., 2012; Sompol et al., 2017, 2021). Here, the use of two-photon imaging showed that cerebrovessel leakiness in CT diet animals was exacerbated by VIVIT, which could lead to later deleterious changes in brain structure/function. Altogether, these effects are generally in the opposite direction of what is observed in disease models where improved synapse function, reduced glial reactivity, and so forth are the norm with AAV-Gfa2-VIVIT treatment. The results suggest that CN/NFAT signaling in astrocytes can play protective roles in healthy astrocytes, which is consistent with recent findings from Lim et al. (2022). Determining when CN/NFAT diverges from its protective properties in the context of astrocyte reactivity should be pursued in future studies.

### Summary

The present findings derived from an HHcy model of VCID further confirm that reactive astrocyte signaling through the CN/NFAT pathway is a common mechanism for synapse dysfunction in aged, injured, or diseased brain. Our data further indicate that reactive astrocytes and CN/NFATs play deleterious roles in functional hyperemia, which could disrupt the coupling between energy demand and supply in the brain. Given the high comorbidity rate between VCID and AD (as well as many other ADRDs), the results suggest that reactive astrocyte signaling in general, and CN/NFAT activity in particular, may offer attractive drug targets for treating a broad swath of individuals with single and multiple etiology dementia.
