# Supplementary figures and images for: Targeting Astrocyte Signaling Alleviates Cerebrovascular and Synaptic Function Deficits in a Diet-Based Mouse Model of Small Cerebral Vessel Disease
Source: J Neurosci. 2023 Mar 8;43(10):1797–813. doi: 10.1523/JNEUROSCI.1333-22.2023 (PMC10010459; doi:10.1523/JNEUROSCI.1333-22.2023)

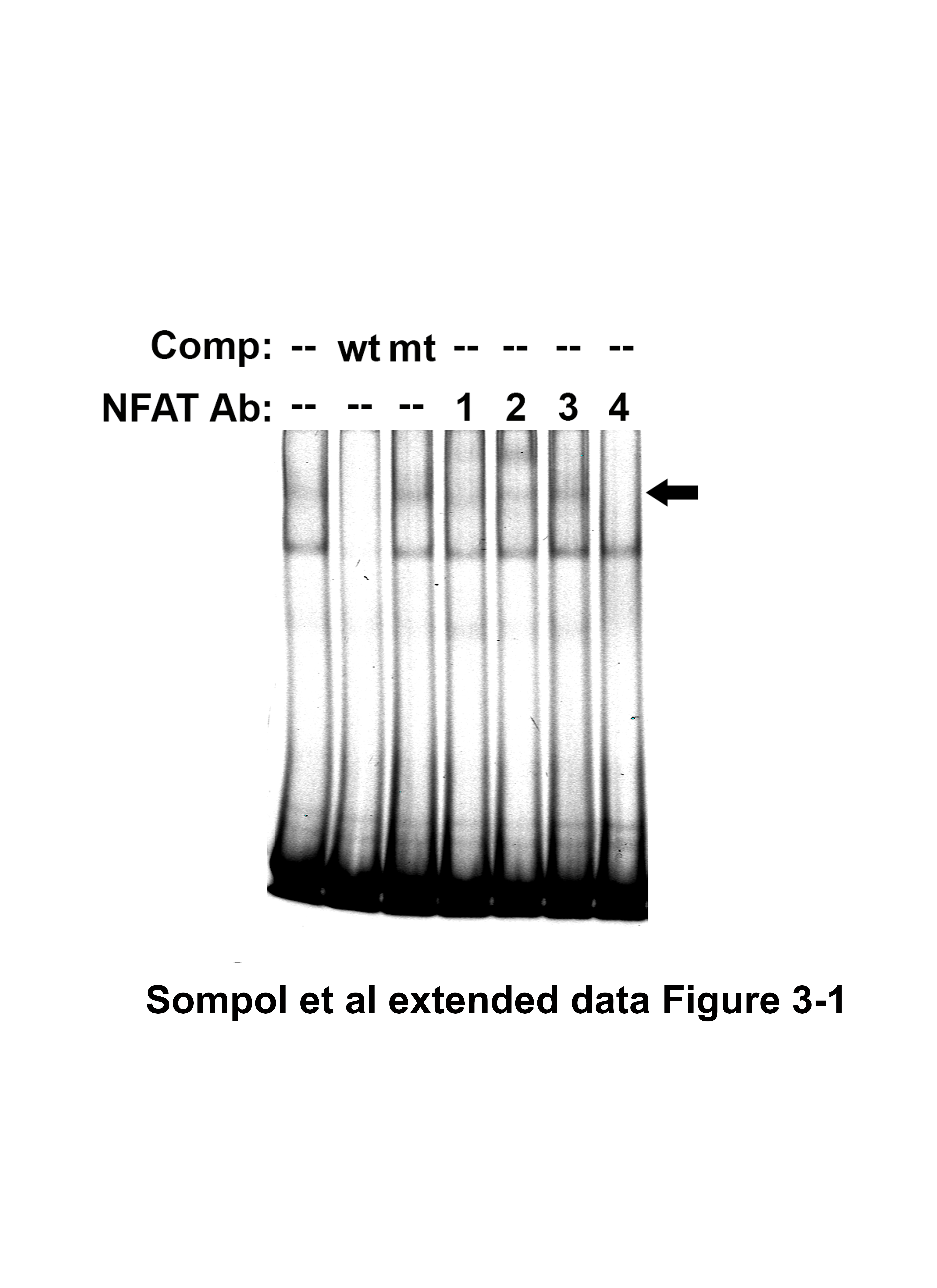

Supplement: Figure 3-1 — Using EMSA to identify NFAT4-DNA complexes in mouse brain tissue. Representative EMSA for brain homogenates from a control diet mouse. NFAT-binding probes were added to homogenates with and without antibodies (Ab) to each of the four CN-dependent NFAT isoforms (1–4) to show supershifts and/or block shifts. Unlabeled WT (wt) and mutant (mt) DNA probe was included in some conditions to demonstrate DNA-binding specificity of the labeled probe. The arrow indicates a clear block shift where the NFAT4 antibody was included with the DNA-binding probe. Download Figure 3-1, TIF file. [file ns-JN-RM-1333-22-s01.tif]

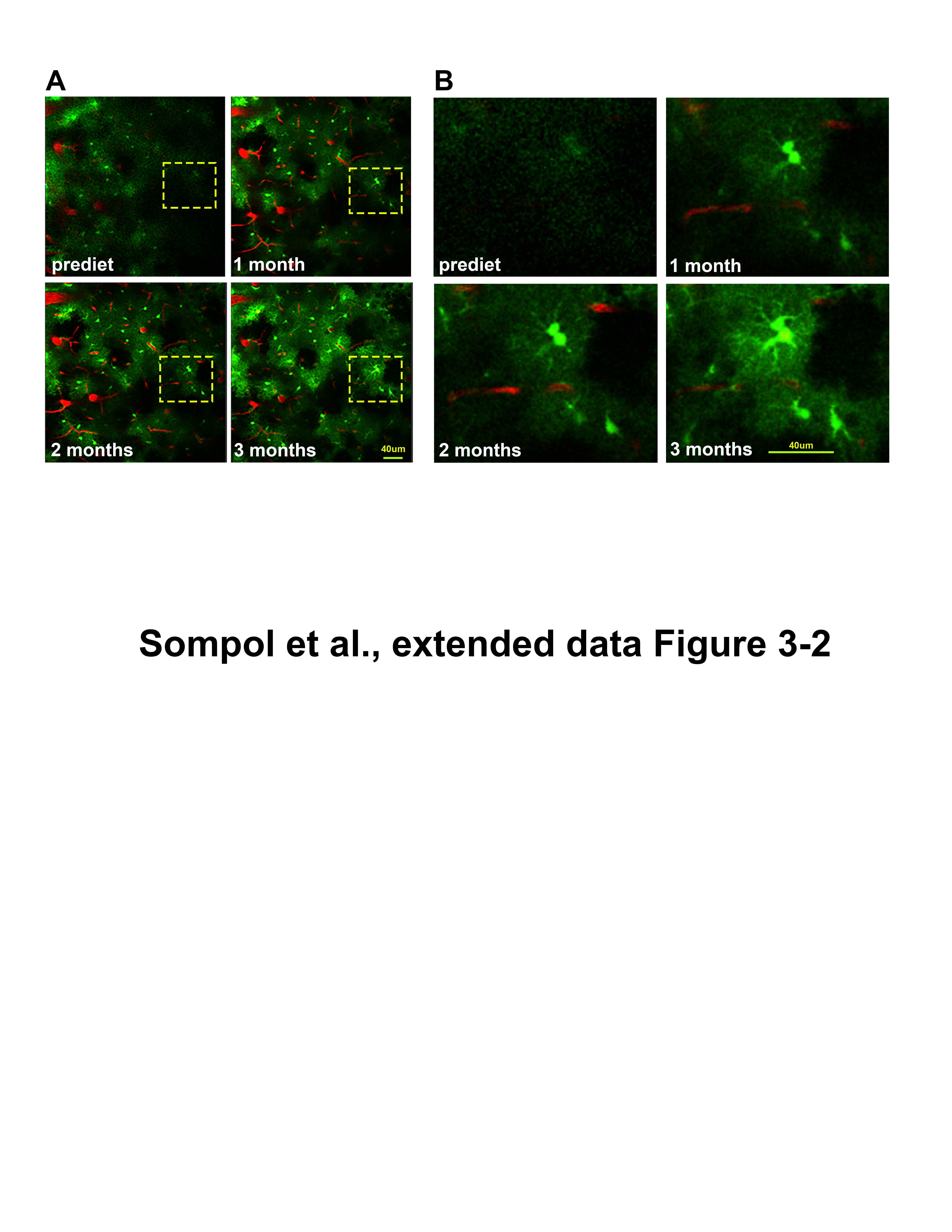

Supplement: Figure 3-2 — Using EGFP expression as an indicator of GFAP promoter activity. A, FOV in barrel cortex of a mouse injected with AAV-Gfa2-EGFP. Scale bar, 40 µm. Two-photon microscopy was used to visualize EGFP-expressing astrocytes (green) and cerebral vessels (red, rhodamine-dextran). Images were taken from the same FOV of the same mouse at prediet and then 1, 2, and 3 months after the initiation of HHcy diet. Scale bar, 40 µm. B, High-magnification two-photon images of the hatched boxes in A. Download Figure 3-2, TIF file. [file ns-JN-RM-1333-22-s02.tif]

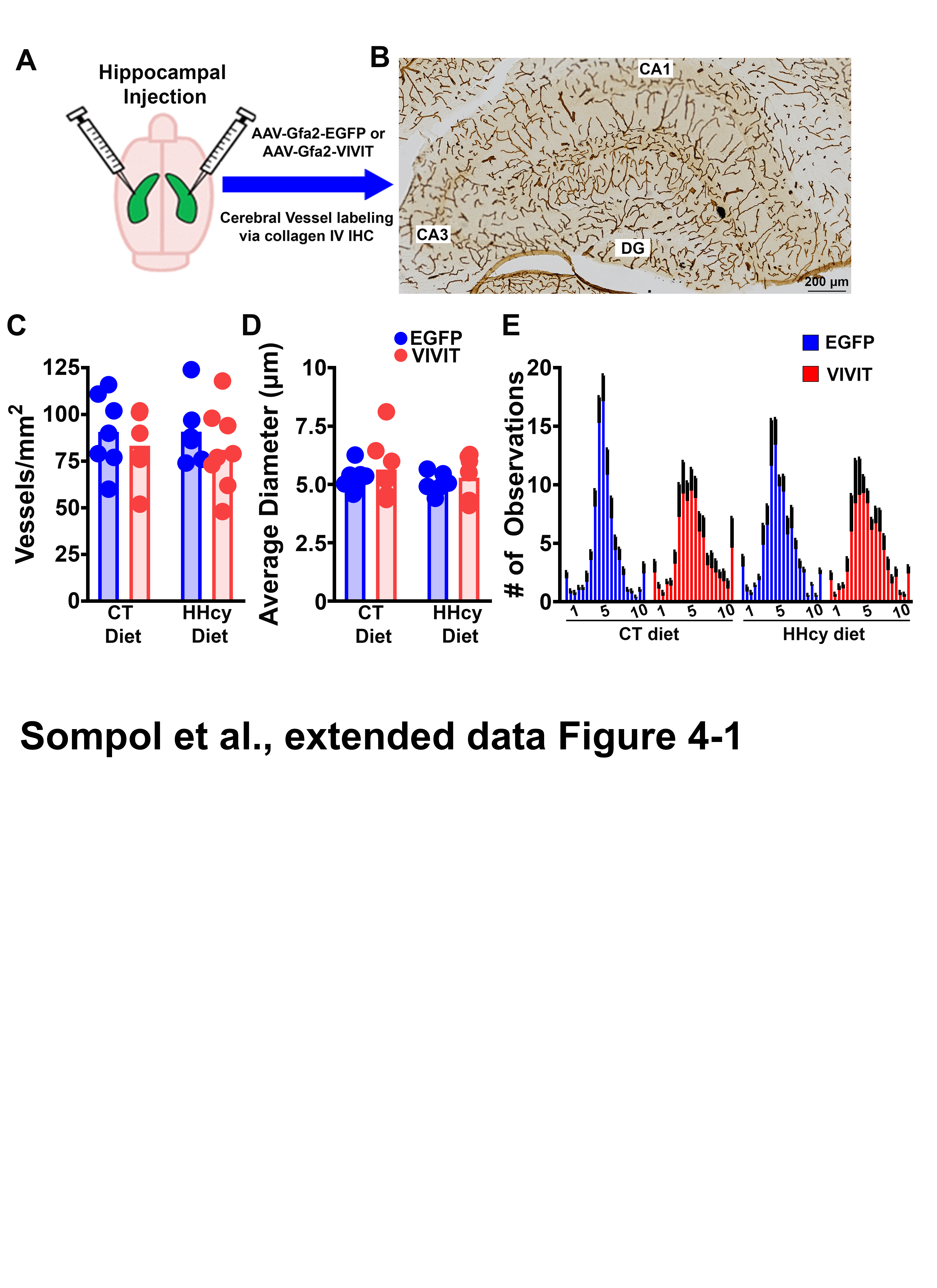

Supplement: Figure 4-1 — Microvessel labeling in diet/AAV-treated mice. A, B, Mice received intrahippocampal injections of AAV-Gfa2-EGFP or AAV-Gfa2-VIVIT. At 1 month after AAV injections, mice were fed for an additional 3 months with CT diet or HHcy diet. Formalin-fixed brain sections were then prepared and labeled for the basement membrane constituent, collagen IV, to reveal microvessels in the hippocampus (B). C, D, Neither the number of labeled vessels per mm2 (C), nor the average vessel diameter (D) was affected by diet or AAV treatment. E, Frequency histograms showing the distribution of microvessel diameters across diet-AAV treatment conditions. Although the distribution showed a higher amplitude in the CT diet-EGFP group, other histogram parameters were similar, and no significant differences were observed across groups using either chi-square tests or Kolmogrov–Smirnov tests. Download Figure 4-1, TIF file. [file ns-JN-RM-1333-22-s03.tif]
